# Flavonoids as Antimicrobial Pesticides: Mechanisms of Action, Formulation Strategies, and Perspectives for Pre- and Postharvest Disease Management

**DOI:** 10.3390/plants15182847

**Published:** 2026-09-17

**Authors:** Verónica Pereira, Lúcia Santos, Paula C. Castilho

**Affiliations:** 1 CQM—Centro de Química da Madeira, Universidade da Madeira, Campus da Penteada, 9200-105 Funchal, Portugal; 2 LEPABE—Laboratory for Process Engineering, Environment, Biotechnology and Energy, ALiCE—Associate Laboratory in Chemical Engineering, Faculty of Engineering, University of Porto, Rua Dr. Roberto Frias, 4200-465 Porto, Portugal; lsantos@fe.up.pt

**Keywords:** flavonoids, antifungal, antibacterial, mechanisms of action, formulation

## Abstract

The global spread and incidence of microbial phytopathogens are expected to increase due to climate change and the intensification of international food trade, threatening crop productivity. While pesticides remain essential for managing disease severity, European regulations and initiatives, such as the Green Deal, have emphasized the urgent need to reduce their indiscriminate use, which has contributed to pest resistance, environmental contamination, and human health issues. Botanical pesticides, particularly flavonoids, represent promising complements to traditional pesticides because of their diverse composition of antimicrobial agents. Therefore, this review provides an overview of the antimicrobial potential of flavonoids for microbial phytopathogens, focusing on agricultural fungal pathogens and bacterial phytopathogens. The mechanisms of action by which flavonoids exert their pesticidal activity are detailed, along with structure–activity relationships, formulation, and market perspectives. The literature reveals that flavonoids possess strong potential against pre- and postharvest crop diseases caused by fungal and bacterial pathogens owing to their ability to exert multisite activity, although limited data is verified for antibacterial studies. However, their successful transition to the pesticide market requires field performance validation, formulation technologies, toxicological assessment, and regulatory harmonization.

## 1. Introduction

Plant diseases induced by pest infections cause an estimated USD 220 billion loss in annual crop productivity [1]. Among the plant pathogens, microbial pathogens contribute significantly to this loss. Phytopathogenic fungi destroy one-third of all food crops annually, and their infection is characterized by spots, blights, and tumor-type glands in plants [2,3]. In addition, these pathogens contribute to 10–20% of postharvest losses [4]. Plant bacterial diseases are estimated at USD 1 billion in losses worldwide every year, and their action leads to abnormal plant growth, spots, blights, and hormone imbalances [2,5].

**Figure 1:**
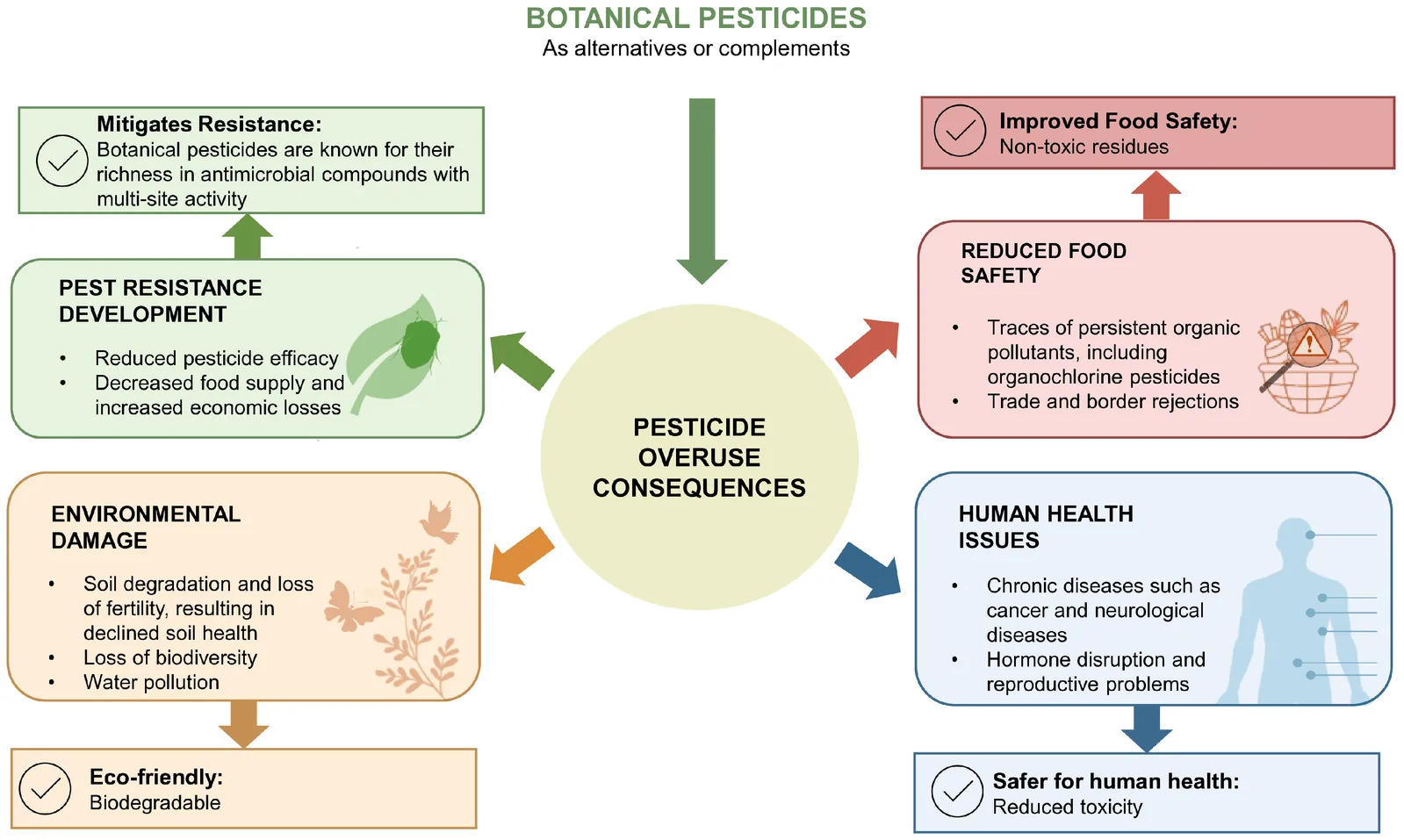
Overview of the major consequences associated with overuse of chemical pesticides and potential benefits of botanical pesticides as alternatives or complements.

Climate change can exacerbate these values because of the intensification of international trade and transport of plants and food products, increasing the proliferation of microorganisms and the appearance of new plant diseases and hosts [1,3,4]. One such case is the global spread of *Fusarium oxysporum* f. sp. *cubense*, which is responsible for Panama disease in banana plants. It was discovered in the 1900s in Java, Indonesia, and is now spread all over the world [6]. Moreover, climate change is characterized by persistent and frequent extreme conditions that put plants under stress (such as drought, extreme temperatures, and salinity), thereby lowering their resistance and defense mechanisms to phytopathogens [1]. These worldwide changes in pathogen distribution and plant resistance pose significant risks to food chain productivity and security, which will affect food reserves and availability for the continuously growing human population. Agrochemicals such as pesticides are essential for securing the food chain supply and maintaining economically viable production for farmers worldwide. However, the European Union (EU) Green Deal brought awareness to the need to decrease the amount of pesticides used because of their well-known harmful effects on human health and the environment (Figure 1) [7,8,9,10,11,12,13,14,15]. This initiative aims to reduce the use and risk of chemical pesticides by 50% by 2030 [16] and reinforces the importance of adopting sustainable and safe approaches in pest control. The use of biopesticides, such as botanical pesticides, in combination with traditional pesticides constitutes a promising measure to achieve this goal.

**Figure 2:**
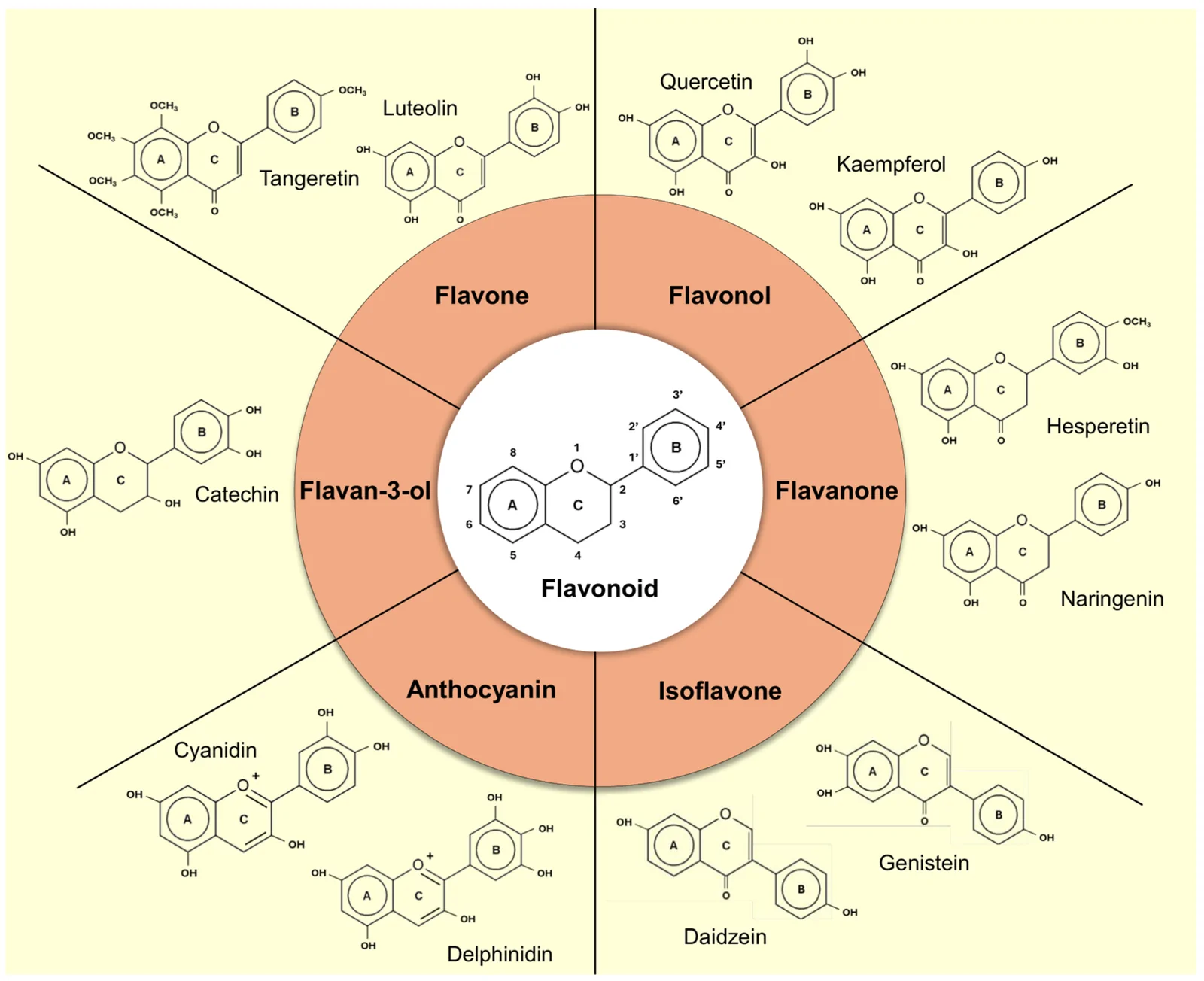
Classification and chemical structures of main flavonoid subclasses. The central structure represents the basic C6-C3-C6 backbone of flavonoids with numbered atoms, surrounded by the subclasses and representative examples.

Botanical pesticides, a subclass of biopesticides, are natural compounds in the form of essential oils and plant-based extracts that control pests via mechanisms non-toxic to humans [17]. Their variety of bioactive compounds has attracted attention as potential microbicides, with reports revealing their capacity to inhibit conidial germination, increase membrane permeability, and lead to abnormal bacterial metabolism [18]. A widely studied class of these compounds is flavonoids (Figure 2), which are secondary metabolites involved in plant tolerance to biotic stress and thus promising candidates for pest disease management [19,20]. For example, *Thymus vulgaris* L. essential oil showed a MIC value of 800 µg/mL against *Agrobacterium tumefaciens* and disrupted the detoxifying reactive oxygen species (ROS) system by downregulating the expression of manganese transport-related genes, affecting the superoxide dismutase activity and, consequently, leading to the accumulation of intracellular ROS and membrane damage [21]. In another study, the essential oil of *Satureja kitaibelii* inhibited 40 to 85% of mycelial growth from three fungal species (*Alternaria alternata, Botrytis cinerea* and *Fusarium oxysporum*) and two oomycetes (*Phytophthora cryptogea* and *Phytophthora nicotianae*), while the exudate extract inhibited the three fungal species (7% for *A. alternata*, 59% for *B. cinerea* and 37% for *F. oxysporum*) and the methanolic extract only inhibited two fungal species (55% for *B. cinerea* and 39% for *F. oxysporum*) [22]. Although botanical pesticides are notoriously recognized for their broad-spectrum antimicrobial activity and environmental friendliness, they have failed as complete replacements for chemical pesticides and are rather seen as complements, allowing for the reduction of their application through synergistic or additive effects to achieve the same level of performance as the traditional pesticide alone. More specifically, botanical pesticides face a lack of standardized extraction methods and the intrinsic variability of the active compound concentration caused by genetic and epigenetic factors, which pose barriers to their regulatory standardization [23]. Additionally, their instability under field conditions can lead to premature degradation, which might be overcome through formulation methods but increases the pesticide production costs. This aspect is particularly relevant for essential oils that are also volatile and highly susceptible to thermal decomposition and auto-oxidation [24]. In contrast, flavonoids (Figure 2), a subclass of polyphenols commonly found in plant extracts, are promising candidates for pest management due to their involvement in plant tolerance to biotic stress, their non-volatile properties and because they are generally less phytotoxic than essential oils [19,20,25].

Although several recent reviews have addressed plant polyphenols as antimicrobial phytochemicals [26,27,28], as well as the antifungal mechanisms of flavonoids against human pathogenic fungi and their modes and mechanisms of action as insecticides [29,30], their specific antifungal and antibacterial activity on pre- and postharvest phytopathogens remains unreviewed. To address this gap, this review summarizes the proposed mechanisms of action of pure and/or isolated flavonoids against pre- and postharvest microbial phytopathogens from pre- and postharvest diseases, explores their structure–activity relationships, and highlights the importance of flavonoid formulation and market perspectives.

## 2. Literature Search Methodology

**Figure 3:**
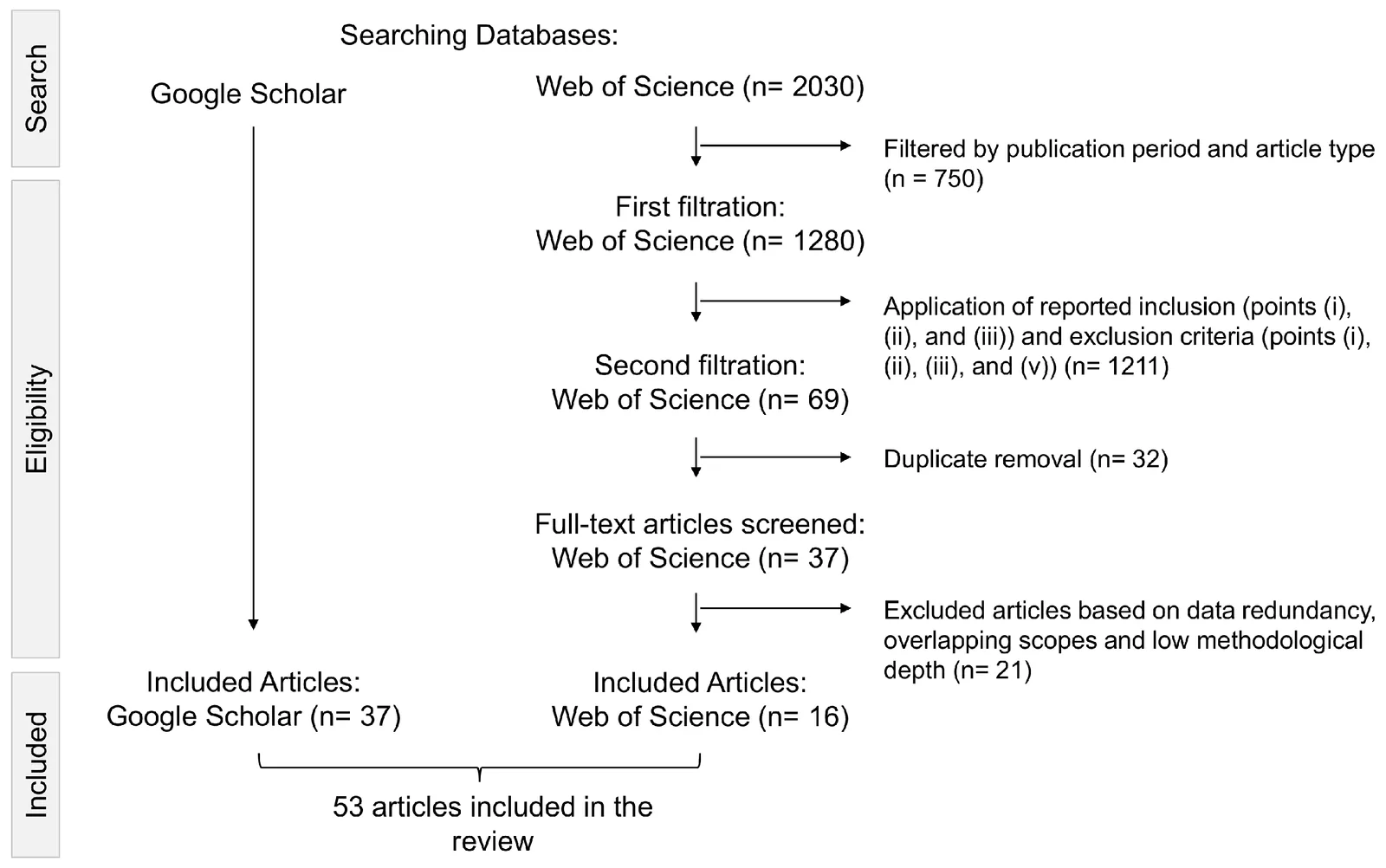
Flow diagram illustrating the literature search, screening, eligibility, and study selection process for the narrative review.

The literature search for original research articles was conducted using electronic databases, primarily Google Scholar and Web of Science Core Collection as a complementary database, alongside Google Patents for identifying patented flavonoids as antimicrobial pesticides. Publications up to December 2025 were considered. Although this review is a narrative review, the search methodology followed a PRISMA-inspired workflow (Figure 3).

The primary search terms were combined using Boolean operators, more specifically “AND”. For Section 3 and Section 4, the keywords included: “flavonoid” AND “antifungal” AND “phytopathogen”, “flavonoid” AND “antibacterial” AND “phytopathogen”, “flavone” AND “antifungal” AND “phytopathogenic”, “flavone” AND “antibacterial” AND “phytopathogenic”, “flavonol” AND “antifungal” AND “phytopathogenic”, “flavonol” AND “antibacterial” AND “phytopathogenic”, “flavanone” AND “antifungal” AND “phytopathogen”, “flavanone” AND “antibacterial” AND “phytopathogen”, “isoflavone” AND “antifungal” AND “phytopathogen”, “isoflavone” AND “antibacterial” AND “phytopathogen”, “flavonoid” AND “antifungal” AND “phytopathogen” AND “mode of action”, “flavonoid” AND “antibacterial” AND “phytopathogen” AND “mode of action”, “flavonoid” AND “antifungal” AND “phytopathogen” AND “mechanism of action”, “flavonoid” AND “antibacterial” AND “phytopathogen” AND “mechanism of action”.

For Section 5, the specific keywords used were: “flavonoid” AND “formulation” AND “antifungal”, “flavonoid” AND “formulation” AND “antibacterial”; “flavonoid” AND “nanoemulsion” AND “antifungal”; “flavonoid” AND “nanoemulsion” AND “antibacterial”; “flavonoid” AND “liposomes” AND “antifungal”, “flavonoid” AND “liposomes” AND “antibacterial”, flavonoid AND “cyclodextrins” AND “antifungal”, flavonoid AND “cyclodextrins” AND “antibacterial”, “flavonoid” AND “nanoparticles” AND “antifungal”, “flavonoid” AND “nanoparticles” AND “antifungal” AND “phytopathogen”, “flavonoid” AND “nanoparticles” AND “antibacterial” AND “phytopathogen”.

The eligibility of studies was evaluated through title and abstract based on defined inclusion and exclusion criteria. As inclusion criteria, original peer-reviewed articles were considered when they: (i) evaluated the antifungal and antibacterial activity of pure flavonoids against agricultural phytopathogens; (ii) provided insights into the modes and mechanisms of action of flavonoids against phytopathogenic fungi and bacteria; or (iii) described formulation studies on flavonoids for field applications. Studies were excluded from the review when: (i) they evaluated flavonoid-rich extracts and other compounds without proper evaluation of individual flavonoid contribution for the reported antifungal and antibacterial activity; (ii) they included non-agricultural pathogens (such as human pathogenic microorganisms and pathogens from processed food products); (iii) formulations or mechanistic studies were not performed with pure flavonoids; (iv) they were non-original and non-peer reviewed original publications (including review articles, conference proceedings, book chapters, preprints); and (v) they were articles published in languages other than English.

Articles presenting contradictory results were not automatically excluded from the review and, instead, were evaluated based on methodological differences and pathogen species susceptibilities.

## 3. Antifungal Agents

Research over the past decades has demonstrated that flavonoids are promising antifungal agents for pest management. As summarized in Table 1, these compounds affect mycelial growth, spore germination, and appressorium formation in numerous fungal pathogens. This activity occurs in a dose-dependent manner and can be related to each flavonoid subclass, demonstrating essential structural features for pronounced inhibitory activity. Moreover, the structural and physiological differences among species also influence flavonoid efficacy. For example, rutin (Table 1) stimulated the germination tube and conidium formation of *A. alternata* across all tested concentrations (1, 5, and 10 mM), while it suppressed germination tube elongation, secondary hyphal branching, and radial growth of *B. cinerea* [31]. However, dose–response effects can be complex and, in some cases, low flavonoid concentrations can induce hormesis effects by stimulating mycelial growth or sporulation rather than inhibiting it [32]. This was also verified for rutin in the same study, which stimulated and inhibited mycelial growth of *Fusarium solani* below 0.1 and above 5 mM, respectively [31]. These low-dose stimulatory responses can trigger adaptive and defensive responses from microorganisms, which can eventually culminate in pathogen resistance. As a result, accurately determining the effective concentration is crucial, especially when translating to in vivo studies, where flavonoids can suffer from premature degradation due to abiotic factors (such as ultraviolet radiation, temperature, and moisture) and higher concentrations might be needed to achieve the desired activity.

Importantly, the literature reveals variable information on flavonoid antifungal efficacy within the same fungal species, which is not always explained by methodological differences. Genetic differences among strains influence membrane composition, permeability, and enzymatic processes, which affect their susceptibility to the same treatment [33]. For instance, naringenin inhibits the growth of *B. cinerea* strains at different levels and had no inhibition effect on species and strains of *Colletotrichum* spp. (Table 1), highlighting the need for strain-specific evaluations and avoiding generalized assumptions [34]. Strain-dependent susceptibility represents a practical limitation in microbial pathogen management, as efficacy against a single strain cannot be broadly generalized to all field strains of the same species. For flavonoid-based antimicrobial pesticides, this aspect evidences the necessity of multisite activity or synergistic formulations for broad-spectrum activity against microbial strains. Despite these biological differences, flavonoids often exhibit multiple mechanisms of action against fungal species, which enhance their antifungal potential and reduce the likelihood of resistance development.

**Table 1:** Antifungal activity of flavonoids against pre- and postharvest phytopathogens.

Flavonoid	Target Organism	Type of Study	Main Results	Commercial Fungicide	Reference
**Anthocyanin**					
Callistephin	*Botrytis cinerea*	In vitro	Significant inhibitory effects on germ tube elongation.	n.a.	[35]
Chrysanthemin	*Botrytis cinerea*	In vitro	Significant inhibitory effects on germ tube elongation (9.76 µm after 24 h) and mycelial growth diameter (14.90 mm after 72 h).	n.a.	[35]
**Flavan-3-ol**					
Catechin	*Botrytis cinerea*	In vitro	Significant inhibitory effects on germ tube elongation (62.37 µm after 24 h) and mycelial growth diameter (6.24 mm after 72 h).	n.a.	[35]
	*Colletotrichum* spp.	In vitro	All tested concentrations (5, 10, and 50 mM) had no effect on inhibiting *Colletotrichum acutatum* (6 isolated), *Colletotrichum gloeosporioides* (2 isolated), *Colletotrichum fragariae*, and *Colletotrichum graminicola*.	n.a.	[36]
**Flavanone**					
Hesperetin	*Phytophthora nicotianae*	In vitro	IC_50_ value for inhibition of mycelial growth was 30.20 mg/L after 7 days.	n.a.	[37]
Homoeriodictyol	*Fusarium graminearum*	In vitro	IC_50_ value for inhibition of fungal radial growth was 274.78 mg/L after 72 h.	Carbendazim (IC_50_ = 2.17 mg/L)	[38]
	*Septoria zeicola*	In vitro	IC_50_ value for inhibition of fungal radial growth was 240.31 mg/L after 72 h.	Carbendazim (IC_50_ = 3.10 mg/L)	
Liquiritigenin	*Phytophthora nicotianae*	In vitro	IC_50_ value for inhibition of mycelial growth was 51.43 mg/L after 7 days.	n.a.	[37]
Naringenin	*Botrytis cinerea*	In vitro	Naringenin (200 mg/L) clustered with pterostilbene reduced the mycelial growth of the strains GBR, GBW, BGM and TGM by 41.2, 36.8, 37.3, and 37.7%, respectively. IC_50_ value for inhibition of fungal growth was 502 ± 7.3 mg/L after 72 h.	n.a.	[34]
	*Colletotrichum* spp.	In vitro	All tested concentrations (5, 10, and 50 mM) had no effect on inhibiting *C. acutatum* (6 isolated), *C. gloeosporioides* (2 isolated), *C. fragariae*, and *C. graminicola*.	n.a.	[36]
	*Magnaporthe oryzae*	In vitro	MIC and IC_50_ values for inhibiting fungus growth were 200 and 102.4 µg/mL, respectively, after 24 h of incubation.	Cycloheximide (MIC = 1.6 μg/mL)	[39]
	*Phytophthora capsici*	In vitro	IC_50_ value for inhibition of mycelial growth was 50.11 mg/L after 7 days.	n.a.	[37]
	*Phytophthora infestans*	In vitro	MIC and IC_50_ values to inhibit fungus growth were 50 and 53.7 µg/mL, respectively, after 24 h.	Cycloheximide (MIC = 1.6 μg/mL)	[39]
	*Phytophthora nicotianae*	In vitro and In vivo	IC_50_ values for inhibition of sporangia production and mycelial growth were 2.01 and 22.01 mg/L, respectively, after 7 days of incubation. Plant treated with naringenin (20 mg/mL) induced the accumulation of H_2_O_2_ and O_2_^−^ and the expression of salicylic acid biosynthesis-related genes.	n.a.	[37]
	*Pyricularia oryzae*	In vitro	Naringenin (7 and 14 µg) significantly inhibited spore germination after 5 h of incubation (9.60 and 1.73% of germinated spores, respectively). Absence of germ tube and appressorium formation.	n.a.	[33]
	*Rhizoctonia solani*	In vitro	No effect on the mycelial or sclerotial growth.	n.a.	
Pinocembrin	*Aspergillus niger*	In vitro	MIC value for inhibition of fungal growth was 32 ± 0.02 µg/mL.	Ketoconazole (MIC = 8 ± 0.01 µg/mL) Clotrimazole (MIC = 3 ± 0.02 µg/mL)	[40]
	*Fusarium* spp.	In vitro	MIC values for inhibition of fungal growth were 45 ± 0.02, 30 ± 0.01, and 30 ± 0.02 µg/mL for isolated from *G. max*, *M. indica*, and *G. max*, respectively.	Ketoconazole (MIC = 2 ± 0.02, 3 ± 0.02, and 3 ± 0.02 µg/mL) Clotrimazole (MIC = 6 ± 0.02, 3 ± 0.01, and 3 ± 0.02 µg/mL)	
	*Phomopsis* sp.	In vitro	MIC value for inhibition of fungal growth was 32 ± 0.01 µg/mL.	Ketoconazole (MIC = 3 ± 0.02 µg/mL) Clotrimazole (MIC = 3 ± 0.01 µg/mL)	
	*Penicillium italicum*	In vitro	Pinocembrin (400 mg/L) inhibited the mycelial growth and spore germination by 93 and 97%, respectively.	n.a.	[41]
Pinocembroside	*Alternaria citri*	In vitro	MIC and MFC values for inhibiting mycelial growth equal to 0.8 g/L after 2 and 6 days of incubation, respectively.	n.a.	[42]
	*Colletotrichum gloeosporioides*	In vitro	MIC and MFC values for inhibiting mycelial growth equal to 0.2 and 0.4 g/L, respectively, after 2 and 6 days of incubation, respectively.	n.a.	
	*Diaporthe citri*	In vitro	MIC and MFC values for inhibiting mycelial growth equal to 0.1 and 0.4 g/L, respectively, after 2 and 6 days of incubation, respectively.	n.a.	
	*Geotrichum citri-aurantii*	In vitro	MIC and MFC values for inhibiting mycelial growth equal to 0.2 and 0.8 g/L, respectively, after 2 and 6 days of incubation, respectively.	n.a.	
	*Penicillium digitatum*	In vitro	MIC and MFC values for inhibiting mycelial growth equal to 0.2 and 0.4 g/L, respectively, after 2 and 6 days of incubation, respectively.	n.a.	
	*Penicillium italicum*	In vitro and In vivo	MIC and MFC values for inhibiting mycelial growth equal to 0.2 and 0.8 g/L, respectively, after 2 and 6 days of incubation, respectively. In vivo test on “Newhall” navel oranges revealed that, in fruits treated with pinocembroside at 4 g/L, the lesion diameter decreased by 94.3% compared with the control. In vitro and in vivo results indicated a dose-dependent activity.	n.a.	
**Flavone**					
Baicalein	*Paecilomyces variotii Bainier*	In vitro	IC_50_ value for inhibiting mycelium growth was 40.05 mg/mL.	n.a.	[43]
	*Poria vaporaria Cooke*	In vitro	IC_50_ value for inhibiting mycelium growth was 12.40 mg/mL.	n.a.	
Luteolin	*Fusarium graminearum*	In vitro	IC_50_ value for inhibition of fungal radial growth was 56.38 mg/L after 72 h of incubation.	Carbendazim (IC_50_ = 2.17 mg/L)	[38]
	*Fusarium oxysporum f.* sp. *lycopersici*	In vitro	Luteolin (25, 50, and 100 µM) showed a low stimulating activity on microconidia germination.	n.a.	[44]
	*Septoria zeicola*	In vitro	IC_50_ value for inhibition of fungal radial growth was 81.48 mg/L after 72 h.	Carbendazim (IC_50_ = 3.10 mg/L)	[38]
Tangeretin	*Aspergillus parasiticus*	In vitro	At 50 µg/mL, approximately 80% of radial mycelial growth was inhibited after 4 days.	Benomyl (100% inhibition at 50 µg/mL)	[45]
	*Colletotrichum gloeosporioides*	In vitro	At 50 µg/mL, approximately 70% of radial mycelial growth was inhibited after 4 days.	Benomyl (100% inhibition at 50 µg/mL)	
	*Fusarium culmorum*	In vitro	At 50 µg/mL, approximately 30% of radial mycelial growth was inhibited after 4 days.	Benomyl (100% inhibition at 50 µg/mL)	
	*Geotrichum candidum*	In vitro	At 50 µg/mL, approximately 30% of radial mycelial growth was inhibited after 4 days.	Benomyl (0% inhibition at 50 µg/mL)	
	*Penicillium italicum*	In vitro	At 50 µg/mL, approximately 35% of radial mycelial growth was inhibited after 4 days.	Benomyl (100% inhibition at 50 µg/mL)	
	*M. oryzae*	In vivo	At 200 µmol/L and higher concentrations, blocked appressorium formation in a reversible mode, indicating that tangeretin interferes with appressorium formation but does not kill conidial cells at least after 8 h of treatment. Rice leaves treated with tangeretin prior to fungus inoculation led to smaller disease lesions. At 200 µmol/L, prevented disease lesions in rice seedlings inoculated with the fungus in the nursery, while some symptoms were verified in the experimental field plots.	Tricyclazole (at 2 g/L, absence of blast symptoms similar to tangeretin in the nursery but presence of symptoms in experimental field plots)	[46]
**Flavonol**					
Galangin	*Aspergillus niger*	In vitro	MIC value for inhibition of fungal growth was 28 ± 0.01 µg/mL.	Ketoconazole (MIC = 8 ± 0.01 µg/mL) Clotrimazole (MIC = 3 ± 0.02 µg/mL)	[40]
	*Fusarium* spp.	In vitro	MIC values for inhibition of fungal growth were 35 ± 0.02, 25 ± 0.02, and 27 ± 0.01 µg/mL for the fungus isolated from *G. max*, *M. indica*, and *G. max*, respectively.	Ketoconazole (MIC = 2 ± 0.02, 3 ± 0.02, and 3 ± 0.02 µg/mL) Clotrimazole (MIC = 6 ± 0.02, 3 ± 0.01, and 3 ± 0.02 µg/mL)	
	*Phomopsis* sp.	In vitro	MIC value for inhibition of fungal growth was 30 ± 0.03 µg/mL.	Ketoconazole (MIC = 3 ± 0.02 µg/mL) Clotrimazole (MIC = 3 ± 0.01 µg/mL)	
Hyperoside	*Botrytis cinerea*	In vitro	Dose-dependent activity, with significant linear inhibitory effects on germ tube elongation (28.18 µm after 24 h) and mycelial growth diameter (9.74 mm after 72 h).	n.a.	[35]
Kaempferol	*Fusarium oxysporum*	In vitro	Higher activity of KAE-LC nanoparticles (300 ppm, 67%) than pure kaempferol (300 ppm, no inhibition) after a 60-day storage period. In nanoparticle-treated cultures, hyphae appeared distorted and fragmented.	n.a.	[47]
	*Pyricularia oryzae*	In vitro	Kaempferol (7 and 14 µg) inhibited spore germination after 5 h of incubation (47 and 35% of germinated spores, respectively).	n.a.	[33]
	*Rhizoctonia solani*	In vitro	No effect on the mycelial or sclerotia growth.	n.a.	
Quercetin	*Colletotrichum* spp.	In vitro	All tested concentrations (5, 10, and 50 mM) had no effect on inhibiting *C. acutatum* (6 isolated), *C. gloeosporioides* (2 isolated), *C. fragariae*, and *C. graminicola*.	n.a.	[36]
	*Fusarium solani*	In vitro	Quercetin (15 mg/mL) completely inhibited mycelial growth after 5 days.	n.a.	[48]
	*Fusarium oxysporum f.* sp. *vasinfectum*	In vitro	IC_50_ value for inhibiting spore germination was 42.4 ± 2.6 µg/mL.	Carbendazim (IC_50_ = 29.5 ± 3.1 mg/L)	[49]
	*Fusarium oxysporum f.* sp. *cucumerinum*	In vitro	IC_50_ value for inhibiting spore germination was 32.9 ± 1.4 µg/mL.	Carbendazim (IC_50_ = 27.6 ± 3.1 mg/L)	
Rutin	*Alternaria alternata*	In vitro	Stimulated the formation of germ tubes after 6 h of incubation. In all tested concentrations (1, 5, and 10 mM), stimulated conidium formation (from 36 h to 48 h of incubation).	n.a.	[31]
	*Botrytis cinerea*	In vitro	At 10 mM, it greatly inhibited the prolongation of germ tubes, secondary hyphal branching, and radial growth after 6 and 24 h of incubation.	n.a.	[31]
	*Fusarium solani*	In vitro	Stimulated the formation of germ tubes but did not significantly affect their prolongation. At concentrations higher than 5 mM, rutin inhibited mycelial growth, while at concentrations lower than 0.1 mM, rutin could stimulate mycelial growth.	n.a.	[31]
	*Fusarium oxysporum f.* sp. *vasinfectum*	In vitro	IC_50_ value for inhibiting spore germination was 357.8 ± 15.5 µg/mL.	Carbendazim (IC_50_ = 29.5 ± 3.1 mg/L)	[49]
	*Fusarium oxysporum f.* sp. *cucumerinum*	In vitro	IC_50_ value for inhibiting spore germination was 257.3 ± 23.42 µg/mL.	Carbendazim (IC_50_ = 27.6 ± 3.1 mg/L)	
Taxifolin	*Fusarium graminearum*	In vitro	IC_50_ value for inhibition of fungal radial growth was 124.27 mg/L after 72 h.	Carbendazim (IC_50_ = 2.17 mg/L)	[38]
	*Septoria zeicola*	In vitro	IC_50_ value for inhibition of fungal radial growth was 160.32 mg/L after 72 h.	Carbendazim (IC_50_ = 3.10 mg/L)	
**Isoflavone**					
Equol	*Magnaporthe oryzae*	In vitro and In vivo	Affected mycelial growth, conidial generation and germination, and appressorial formation Reduced fungal virulence on rice and barley leaves.	n.a.	[50]
Daidzein	*Poria vaporaria Cooke*	In vitro	IC_50_ value for inhibiting mycelium growth was 40.04 mg/mL.	n.a.	[43]

Note: ${\mathrm{IC}}_{50}$ (half-maximal inhibitory concentration), MIC (minimum inhibitory concentration), ${\mathrm{EC}}_{50}$ (half-maximal effective concentration), and MFC (minimum fungicidal concentration), n.a. (not applicable). Concentration units are reported as mass (mg/L, μg/mL, mg/mL), molar (μM), or percentage (%) values. For direct comparison: 1 mg/L=0.001 mg/mL; conversions between mass-based and molar concentrations depend on the molecular weight of each individual flavonoid compound.

### 3.1. Antifungal Modes of Action

#### 3.1.1. Effects on Cell Wall

**Figure 4:**
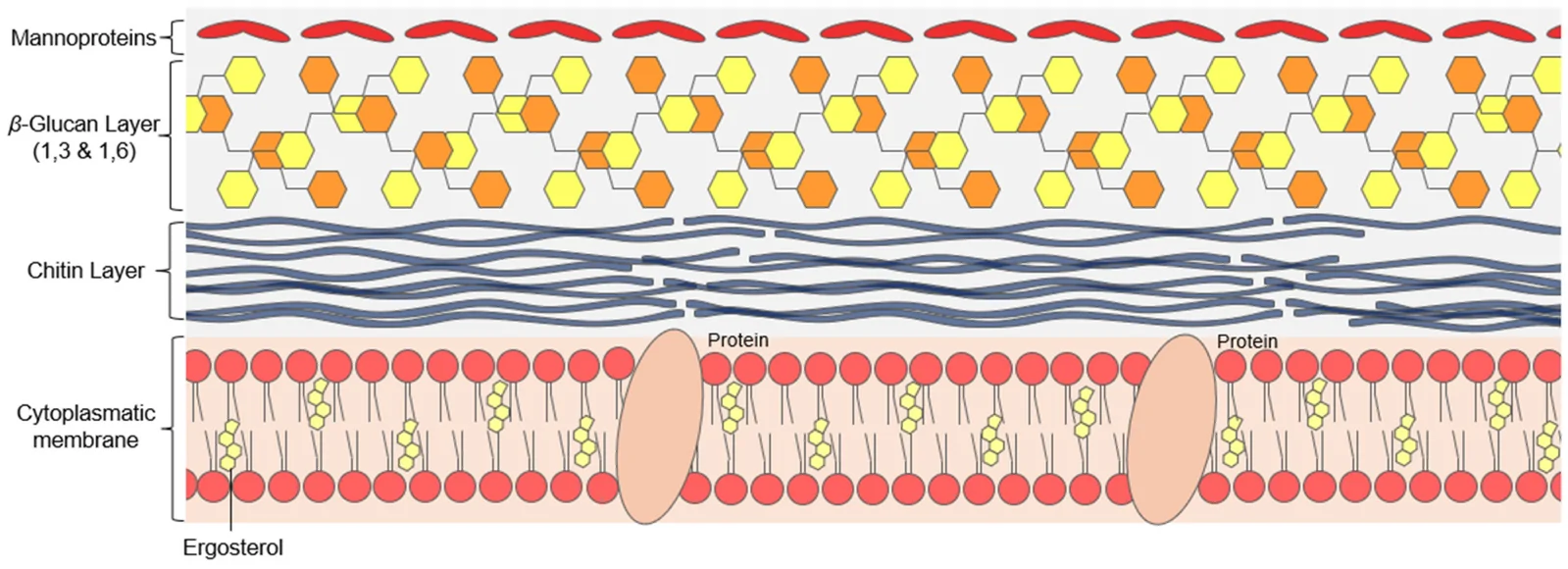
Structural organization of the fungal cell wall and membrane.

The fungal cell wall (Figure 4) plays an important role in protecting cells from osmotic and mechanical stress, controlling permeability and interactions with the external environment [51]. This layer is an ideal target for fungicide development because its polymeric composition is specific to fungi, allowing selective fungicide action while minimizing the risk of unintended toxicity to humans and most plants [52].

Glycoproteins, such as mannoproteins, compose the flexible and outer layer of the cell wall and account for 20 to 30% of the dry weight of the wall of filamentous fungi [51]. These proteins are usually coupled to N- and O-oligosaccharides by the addition of mannose residues by mannosyltransferases and appear to be associated with cellular development and processes such as conidial separation, germination, and appressorium formation, as well as osmotic and cell wall stress and virulence [53]. The deletion or suppression of genes encoding *O*-mannosyltransferase and *N*-mannosyltransferase has been the aim of research in several species, such as *Fusarium oxysporum* f.sp. *cucumerinum* [54], *Colletotrichum fructicola* [55], and *Magnaporthe oryzae* [56,57]. These genetic studies demonstrate that the loss of mannosyltransferase activity impairs fungal development (by interfering, for instance, with cell wall integrity and hyphal morphology) and pathogenicity, highlighting mannosyltransferases, especially *O*-mannosyltransferases, as viable targets for fungicide development. Unlike *N*-mannosyltransferases, which are also present in plants and nematodes, *O*-mannosyltransferases have not been identified in these organisms, but only in fungi. This absence significantly reduces the risk of unintended toxicity, making *O*-mannosyltransferases more selective and safer fungicide targets. To the best of our knowledge, the effects of flavonoids on these enzymes have not been considered or evaluated.

The more rigid and mobile inner layer of the cell wall is mainly composed of glucans, particularly β-glucans with 1,3 linkage glucose units, which account for 65 to 90% of the total cell wall glucans and are synthesized by transmembrane glycosyl transferases, called glucan synthases [51,52]. Another important polysaccharide located in this layer is chitin, representing 10 to 20% of the dry weight of the filamentous fungi cell wall and synthesized by chitin synthase [51]. Both polysaccharides provide structure to the cell wall and resistance to enzymatic degradation [52], which makes glucan and chitin synthases natural targets for fungicide development. Notably, naringenin could be an effective chitin synthase inhibitor for *Phytophthora infestans* by establishing hydrogen bonds with key amino acids such as Trp539, a direct interaction well-known for chitin biosynthesis inhibitors, although this study was conducted with an analog structure of that of *P. infestans* [39]. Furthermore, naringenin showed a −7.6 kcal/mol binding energy and a ligand efficacy of 0.38 for this enzyme, which were values close to those of the positive control (−8.6 and 0.43 for cycloheximide, respectively). However, the authors failed to validate the computational predictions through in vitro inhibition assays on chitin synthases from this fungal species. Consequently, unconfirmed molecular docking reports should be interpreted only as predictive mechanisms rather than definitive evidence.

In an in vitro study, *Penicillium italicum* mycelia treated with pinocembroside at IC_50_ (0.08 g/L) and MIC (0.2 g/L) values presented 1.13- and 1.36-fold higher β-1,3-glucanase activity, respectively, than the negative control after 6 h of incubation [42]. While the chitinase activity remained stable in the negative control, the enzyme activity increased slowly after 1 h of treatment at the IC_50_ and MIC values, suggesting that the flavonoid can affect the normal function of cell wall enzymes. This was also evidenced by the lower content of chitin in pinocembroside-treated mycelium (around 35 mg/g for control, 18.48 ± 1.33 mg/g at IC_50_ value, and 13.63 ± 2.89 mg/g at MIC value) and of glucan (634.9 ± 22.6 mg/g for control, 304.1 ± 11.8 mg/g at IC_50_ value, and 228.9 ± 19.1 mg/g at MIC value). Zhang et al. (2018) [58] demonstrated in vivo that quercetin treatment (0.25 mg/mL) of kiwifruit infected with *Penicillium expansum* resulted in enhanced enzymatic activity of the fruit’s chitinase and β-1,3-glucanase compared to those of kiwifruit only treated with quercetin or inoculated with the pathogen. These observations suggest that flavonoids can also enhance fruit resistance to fungal infection by promoting the expression of defense-related enzymes that degrade fungal cell wall components [58]. Consistent with this mechanism, an in vivo study demonstrated that a celery flavonoid-rich extract, primarily composed of apigenin, reduced disease severity by 97% at 4 mg/mL caused by *Podosphaera fusca* in cucumber leaves, and induced the upregulation of genes related to defense proteins, including β-1,3-glucanase and chitinase, resulting in high levels of these enzymes [59].

Melanin is another component that can be found within the cell wall but the distribution and abundance vary largely between species. Melanin can be detected in the outer regions of the cell wall, clustered at the surface and/or throughout the cell wall. This pigment is recognized for contributing to fungal virulence and for improving resistance to extreme environmental conditions [51,60]. These properties make the enzymes of melanin biosynthesis pathways a relevant target for fungicide research. Particularly, naringenin was reported to act as a competitive inhibitor of two enzymes from the melanin biosynthetic pathway through in silico studies: 1,3,8-trihydroxynaphthalene reductase and scytalone dehydratase of *P. infestans* [39]. Molecular modeling studies revealed that naringenin binds to a hydrophobic pocket within the active site of 1,3,8-trihydroxynaphthalene reductase, where it is shielded from the exterior. In the case of scytalone dehydratase, naringenin formed hydrogen bonds with key amino acids essential for enzyme inhibition. The authors also suggested that the naringenin skeleton could be a potential lead for fungicide discovery, as the ligand efficiency for both enzymes was 0.43 and 0.46, respectively, similar to the positive control (0.42 and 0.45 for cycloheximide, respectively). Nonetheless, these predictive mechanisms were not confirmed through in vitro enzymatic studies to accurately verify whether naringenin was a competitive inhibitor of these two enzymes.

#### 3.1.2. Effects on Membrane Integrity and Components

Fungal membrane cells are predominantly composed of lipids and sterols, which, in addition to other physiological functions, allow for the permeation of large molecules and maintain the cell water potential [61]. In most studies, phytopathogenic fungi treatment with flavonoids eventually leads to membrane damage by altering its permeability and structure, resulting in cell content leakage. To evaluate membrane damage, several simple to more complex analytical techniques are used. Spectrophotometric assays (for example, for measuring electrolyte leakage, malondialdehyde (MDA) content, and reactive oxygen species (ROS)) are the most commonly reported primary assays, owing to their cost-effectiveness and rapid results. Fluorescent dyes (such as propidium iodide) allow direct visualization of membrane permeability and cell viability but are dependent on staining conditions. Finally, electron microscopy techniques provide detailed high-resolution images of cell morphology but do not allow real-time monitoring, require complex sample preparation, and are expensive. Collectively, these conventional methods do not allow an in-depth determination of the exact mechanisms of action and cannot confirm whether membrane damage is a primary cause or a secondary effect, which is typically achieved through transcriptomics analysis.

In vitro studies reported that the flavanones pinocembrin and its 7-glucoside altered normal cell morphology, increased cell membrane permeability and decreased the intracellular constituent contents of *P. italicum* mycelia when compared to the control [41,42]. In an in vivo study, 1.5% chitosan–catechin coating of post-harvested satsuma oranges resulted in morphological changes in spores of *Penicillium citrinum* and *A. niger*, indicating membrane damage [62]. These physiological changes can be a result of these polyphenols targeting the synthesis and/or degradation of membrane components, more specifically, lipids.

The literature search showed common correlations between the antioxidant and antifungal activities, with the authors speculating that higher antifungal activity was induced by an equally high radical scavenging activity. These assumptions may not always be correct as it has been shown that flavonoids can also act as prooxidants. This behavior can contribute to an accelerated degradation of polyunsaturated fatty acids in the membranes, a phenomenon known as lipid peroxidation and that can lead to cell death. For example, Wang et al. (2022) studied the in vitro antifungal activity of a flavonoid fraction from *Sedum aizoon* L., mainly composed of quercetin and kaempferol, against *B. cinerea* [63]. The flavonoid-treated group showed the accumulation of reactive oxygen species (ROS) and malondialdehyde (MDA), an important marker of lipid peroxidation. The authors also verified that the membrane permeability of the flavonoid-treated group was damaged, owing to the higher content of extracellular macromolecules over time. The mechanism of action underlying this activity may have involved the upregulation and downregulation of genes involved in redox processes and also in the glycerolipid metabolism pathway [63]. In another in vitro study, the glabrindin-treated mycelia of *Sclerotinia sclerotiorum* showed a higher accumulation of endogenous ROS than the untreated mycelia, which may have changed membrane permeability and induced extracellular electrolyte leakage [64]. It was also observed that the mitochondrial membrane potential was lowered by affecting the expression levels of phosphatidylserine decarboxylase. Additionally, glabrindin was reported to act on the ergosterol synthesis-related enzymes of *Fusarium graminearum*, leading to destruction of cell membrane integrity and, consequently, affecting the normal transmembrane transport and membrane potential in vitro [65]. Liang et al. (2021) [46] investigated the in vivo mechanism underlying the activity of tangeretin against appressorium formation of *M. oryzae* in rice seedlings under laboratory conditions. The data showed that the antioxidant property of tangeretin interfered with conidial cell death, which is highly important for *M. oryzae* pathogenicity. Tangeretin suppressed conidial ferroptosis by targeting lipid peroxidation mediated by the NADPH oxidases Nox1 and Nox2 [46].

#### 3.1.3. Effects on Respiration

Aerobic and anaerobic respiration in fungi is a crucial biochemical process for energy production; therefore, inhibitors of the fungal respiratory chain have attracted attention in the design of new fungicides [66]. In the case of flavonoids, these polyphenols can affect mitochondrial respiration. For example, gnaphaliin A reduced oxygen consumption in germinating conidia of *B. cinerea* in vitro, which may have been related to its prooxidant activity and consequent production of ROS in the mitochondria [67]. In another study, pinocembrin interfered in vitro with the energy metabolism of *P. italicum* mycelia, which showed lower contents of adenosine triphosphate (ATP), adenosine diphosphate (ADP), and adenosine monophosphate (AMP) in flavonoid-treated groups than in the control group [41]. In vitro, glabrindin-treated mycelia from *F. graminearum* revealed a lower respiration rate with increasing time [65]. Overall, experimental studies evaluating direct effects on fungal respiration remain relatively scarce compared to membrane integrity studies, highlighting fungal energy metabolism as an underexplored target for mechanistic research.

#### 3.1.4. Effects on Amino Acids and Proteins

Proteins are macromolecules essential for life, as they are involved in important biological processes, such as signal transduction and catalysis of biochemical reactions [61]. Through in silico studies, Júnior et al. (2014) hypothesized that the mechanism underlying the fungicidal activity of chrysin and rutin against *Aspergillus ochraceus* was based on their interaction and binding properties with a protein kinase [68]. Nonetheless, the effect of these two flavonoids on protein kinase was not confirmed through in vitro experiments, remaining a predictive mechanism. In an in vitro study, a flavonoid fraction from *Sedum aizoon* L. upregulated the genes involved in the degradation of three amino acids, suggesting interference with amino acid and protein metabolism [63]. In silico studies have shown that naringenin inhibits the calcium-binding protein calmodulin, whose structure is similar to that of *P. infestans*, which could reduce the ability of fungi to infect plants [39]. The flavanone exhibited a binding energy and ligand efficiency of −5.2 kcal/mol and 0.26, respectively, similar to those of the positive control (−4.9 kcal/mol and 0.25 for cycloheximide, respectively), but this efficacy was not confirmed through in vitro studies. More recently, aldehyde dehydrogenase was advocated as a potential target for the research and development of fungicides, given that the in silico studies revealed that the suppression of the genes FvALDH-43 and FvALDH-96 in *Fusarium verticillioides* affected the pathogenicity and resistance to low-temperature stress [69]. In vitro and in vivo experimental studies were conducted against *F. verticillioides* to verify this hypothesis and taxifolin was confirmed to act as an inhibitor of this enzyme.

#### 3.1.5. Effects on DNA

Deoxyribonucleic acid (DNA) determines the development and reproduction of animals, plants, and microorganisms. DNA-modifying enzymes are responsible for modifying the structure or sequence of DNA through replication, repair, recombination, and transcription [70]. The inhibition of these key enzymes compromises DNA integrity, leading to cell death. Little is known about the effects of flavonoids on the DNA-modifying enzymes of phytopathogenic fungi, being one of the least investigated targets. However, naringenin and naringin could be potential inhibitors of DNA topoisomerase type II in *Ustilago maydis* because they interact with the active site of the enzyme in the same pattern as the reference compounds [71]. Nevertheless, in vitro confirmation studies were not performed to accurately determine the flavanones’ mechanisms of action.

#### 3.1.6. Multisite Activity

The literature review indicated that flavonoids could exhibit fungicide activity through the combination of different mechanisms of action simultaneously. For instance, the prenylflavonoid isoxanthohumol, found in hops and beer, may damage *B. cinerea* mycelium in vitro by increasing electrical conductivity in a dose- and time-dependent manner, enhancing intracellular glycerol content and inducing the production of MDA and hydrogen peroxide [72]. Isoxanthohumol also affected the total carbohydrate content, dehydrogenase activities, and citric acid content, which may have led to metabolic malfunctions owing to the inhibition of the citric acid cycle. Isoxanthohumol-treated mycelia revealed low ATP content, reduced adenosinatrifosfatase activity, and inhibition of cell respiration when compared to the control [72]. In an in vitro study, the prenylated flavonoid kurarinone, isolated from the roots of *Sophora flavescens*, exhibited an EC_50_ value of 16.55 µg/mL against *B. cinerea* and disrupted the cell wall components by affecting the synthesis of chitinase, interfered with normal cell membrane integrity and permeability by promoting leakage of macromolecules and inducing the accumulation of ROS, and affected the energy metabolism by affecting the normal activity of enzymes from the tricarboxylic acid, reducing ATP production [73]. Other examples of potential multisite activity were discussed in the previous sections [39,63,64,65]. These observations are desirable in pesticide research because fungicides with multisite targets lower the possibility of pests becoming resistant.

In some cases, this multisite activity may also contribute to inducing plant resistance by upregulating the genes associated with the defense mechanisms. For instance, naringenin showed an IC_50_ of 22.01 mg/L and 2.01 mg/L for inhibiting the mycelial growth and sporangia production of *Phytophthora nicotianae* in vitro, respectively [37]. In tobacco plants, naringenin induced the accumulation of ROS and the expression of the salicylic acid biosynthesis-related genes, and increased plant resistance by enhancing the expression of genes PR1 and SAR8.2. Moreover, the exogenous application of naringenin enhanced the tobacco seedlings’ resistance to *P. nicotianae* [37].

### 3.2. Structure–Activity Relationship

**Figure 5:**
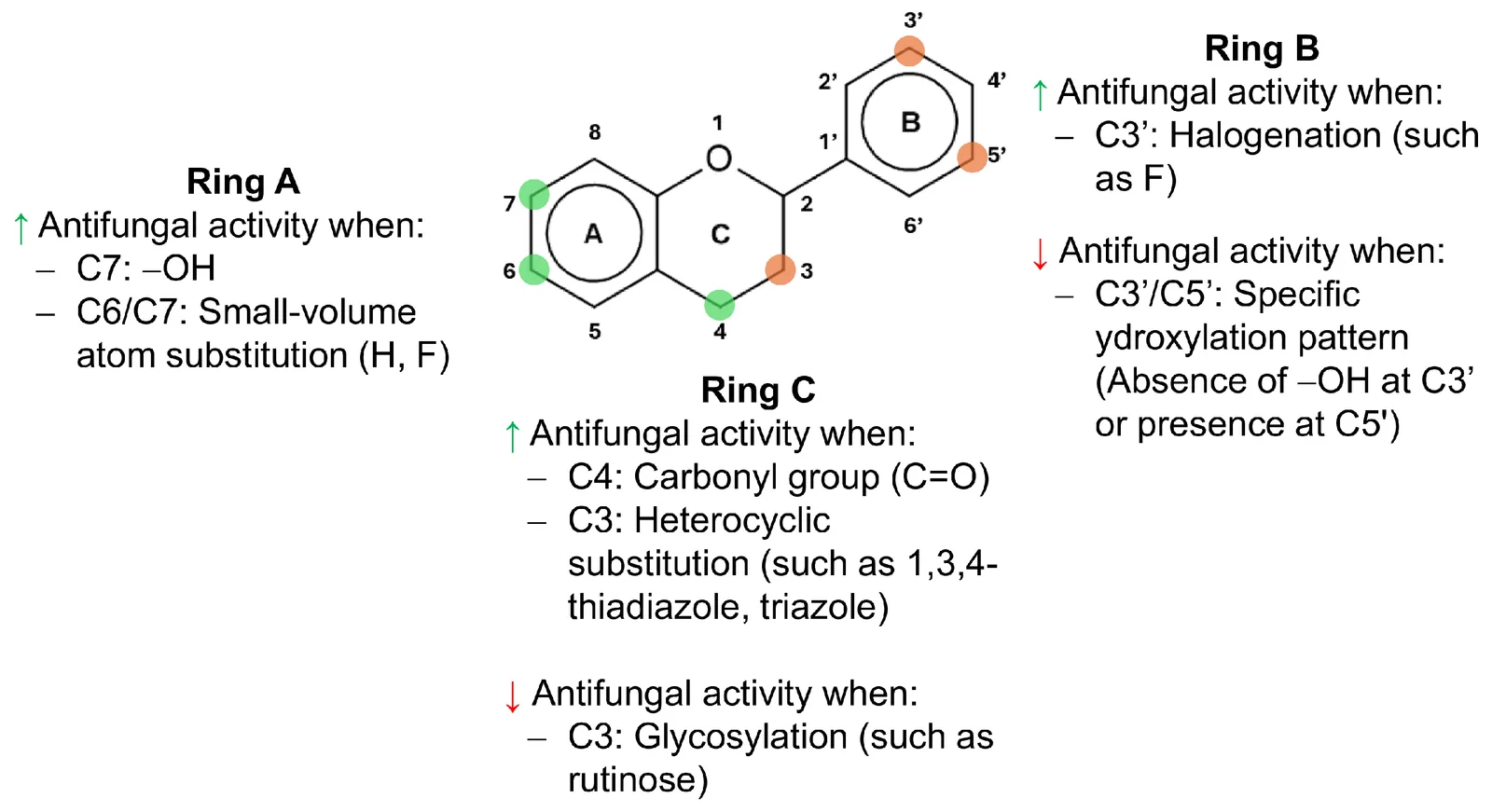
Structure–activity relationships (SAR) of native and modified flavonoids against phytopathogenic fungi. Symbols demonstrate the impact of specific substitutions on antifungal activity (↑: enhanced activity; ↓: reduced activity).

Identifying the functional groups and structural characteristics of flavonoids responsible for their antimicrobial activity is critical for developing and designing improved antimicrobial agents. The nature, number, and position of substituents on the flavonoid skeleton directly influence their interactions and target-binding capacity to fungal cell wall and cell membrane components. Although many studies did not attempt to describe the proper mechanism of action in both studies, differences in the identification of key functional groups for pronounced activity may differ, which can be explained by the distinct cell wall composition of the fungal species involved. This reinforces the notion that structure–activity relationships (SAR) are highly dependent on the pathogen, highlighting the necessity of performing mechanistic studies, such as molecular docking or target-based assays, to clarify how structural features of flavonoids modulate their antifungal activity against specific pathogens. Despite this, these SAR dynamics are usually driven by specific chemical patterns, including hydroxylation, glycosylation, lipophilicity, prenylation, halogen and synthetic heterocyclic substitutions (Figure 5).

The presence, number, and position of hydroxyl and carbonyl groups influence membrane interactions and hydrogen bonding with fungal cell and membrane components. As demonstrated by Ouyang et al. (2017), baicalein exhibited remarkable inhibitory effects against *Poria vaporaria*, followed by dihydromyricetin and daidzein [43]. These three molecules possess two common structural features, namely the carbonyl group at position C4 and a hydroxyl group at C7, which facilitate hydrogen bonding with fungal cell wall and membrane components, contributing to higher antifungal activity. However, hydroxyl substitution on ring B significantly affects the antifungal activity. Evaluation of quercetin, rutin and isoorientin against *F. oxysporum* f. sp. *vasinfectum* and *Fusarium oxysporum* f. sp. *cucumerinum* revealed that the absence of hydroxyl groups at position C3′ and presence of a hydroxyl group at C5′ reduce antifungal efficacy, demonstrating how modifications in ring B alter activity across different fungal targets [49]. Although glycosylation enhances aglycone aqueous solubility, this sugar moiety is reported to decrease antifungal activity. Quercetin exhibited significantly higher antifungal activity than its glycosylated forms, namely isoorientin and rutin. The presence of bulky sugar moieties increases steric hindrance and lowers lipophilicity, restricting passive membrane permeability and reducing direct cellular damage.

Chemical modification of the flavonoid structure represents an essential strategy for the design of new flavonoid-based antimicrobial agents and identifying structural positions prone to chemical modification for enhanced activity. In this regard, heterocyclic substitutions are commonly reported for improved antifungal activity. For example, Zhou et al. (2024) evaluated twenty-two flavone derivatives containing 1,3,4-thiadiazole substitutions against ten phytopathogenic fungi [74]. These heterocyclic compounds are known for their various biological activities, including antifungal and antibacterial properties. The most active compound against *B. cinerea* had hydrogen atoms at the C7 position and in the 1,3,4-thiadiazole ring, coupled with a fluorine atom at the C3′ position. This combination likely minimized steric hindrance, favoring a suitable molecular conformation for target interaction and contributing to compound stability. Further studies indicated that this compound induced the production of endogenous ROS and the release of MDA, leading to membrane lipid peroxidation and, consequently, the release of cell contents.

Similarly, the introduction of nitrogenous and hydrophobic groups, particularly 1,2,4-triazole Schiff base substitutions at position C3, seemed to affect and even increase antifungal activity [75,76]. For example, the evaluation of twenty-four flavonol derivatives containing 1,2,4-triazole Schiff base substitutions against ten filamentous fungi revealed that the flavonol derivative with the highest antifungal activity against *B. cinerea* possessed a hydrogen atom at C7, a 2-methylpropane group at C4′, and a methoxyl group substitution in the benzene ring of the 1,2,4-triazole moiety. As a result, the presence of lipophilic alkyl and methoxyl groups maximized antifungal activity by likely disrupting the cell membrane, which was confirmed by morphological analysis, fluorescence microscopy, cytoplasmic leakage assays, MDA and relative conductivity measurements [75]. In another study, the most active acethydrazide-containing flavonol derivative (substitution at position C3) against *R. solani* possessed electronegative groups at position C4 on the benzene moiety (EC_50_ of 0.170 μg/mL) and strongly inhibited succinate dehydrogenase compared to the fungicide boscalid (IC_50_ = 8.42 and 15.6 μM, respectively) [77].

## 4. Antibacterial Agents

Research on the antibacterial potential of flavonoids has revealed that these polyphenols may serve as promising candidates for managing phytopathogenic bacteria. As summarized in Table 2, flavonoids exhibit variable minimum inhibitory concentration (MIC) values against several economically important bacterial species, with some values exceeding the maximum tested limits. The literature reveals that flavonoid-rich extracts are more typically screened for antibacterial activity than singular compounds. For example, anthocyanin-rich extracts from different berries (blueberry, raspberry, strawberry, and blackberry) showed a MIC value of 6.5% against *Pseudomonas aeruginosa* and *Clavibacter michiganensis* subsp. *michiganensis*, and a lower minimum bactericidal concentration (MBC, 12.5% of extract) was observed for the strawberry and blackberry extracts against all the studied species [78]. In another study, a polymethoxyflavone-rich ethyl acetate extract from orange peel (152.07 mg/g) obtained through ultrasound-assisted extraction exhibited the lowest MIC value (0.156 mg/mL) among the tested extracts against *Xanthomonas citri* subsp. *citri* [79]. In addition to these studies, flavonoids have also been studied for their chemotactic effects on *Ralstonia solanacearum*, but they showed no effect [80].

Notably, flavonoids have been studied less extensively as antibacterial agents than as antifungals, resulting in fewer reports proposing their modes and mechanisms of action and structure–activity relationships. This disparity might be justified by the broader diversity and dispersity of fungal phytopathogens worldwide, which has led to a higher volume of research. On the other hand, antibacterial studies remain more restricted to specific species, which might be related to strict biosafety regulations and quarantine constraints to ensure pathogen containment (for instance, regulated strains of *Xanthomonas* and *Pseudomonas* spp.). However, these polyphenols have been reported (Table 2) to act through multiple mechanisms of action by interfering with the bacterial envelope, impairing biofilm formation, and energy metabolism, making them interesting candidates for bacterial management [81].

**Table 2:** Antibacterial activity of flavonoids against pre- and postharvest phytopathogens.

Flavonoid	Target Organism	Type of Study	Main Results	Commercial Bactericide	Reference
**Flavanones**					
Carthamidin	*Ralstonia solanacearum*	In vitro	Showed an inhibition zone diameter of 8.34 ± 0.16 mm.	Streptomycin sulfate (16.80 ± 0.33 mm)	[82]
Hesperidin	*Xylella fastidiosa*	In vitro and In vivo	In vitro, cis-[Mg(hesp)_2_(phen)]OAc complex was more active (MIC = 1.4 μM) than hesperidin (MIC = 3.3 μM). In vivo, hesperidin reduced bacterial cells to 16.99% of initial abundance, showing moderate activity.	Azadirachtin (MIC = 2.1 μM and reduced bacterial cells to 1.83% in vivo)	[83]
Liquiritigenin	*Ralstonia solanacearum*	In vitro	Showed an inhibition zone diameter of 12.23 mm.	Streptomycin sulfate (16.80 ± 0.33 mm)	[82]
Naringenin	*Xylella fastidiosa*	In vitro and In vivo	In vitro, [Ru(narin)(phen)_2_]PF_6_ and cis-[Mg(narin)(phen)_2_]OAc were more active complexes(MIC 0.19 and 34 μM, respectively) than non-complexed naringenin (7.3 μM). In vivo, naringenin (15.63%) was less active than the respective ruthenium and magnesium complexes (0.12 and 0.65% of initial abundance).	Azadirachtin (MIC = 2.1 μM and reduced bacterial cells to 1.83% in vivo)	[83]
Sophoraflavanone G	*Streptomyces scabiei*	In vitro	At 100 µM, bacterial cell growth was inhibited by 93%. MIC and LD_50_ values of 6.8 ± 0.4 and 2.0 ± 0.1 µM, respectively.	Novobiocin (used to establish the 0% growth baseline)	[84]
**Flavanonol**					
Taxifolin	*Clavibacter michiganensis* subsp. *sepedonicus*	In vitro and In vivo	The diameter of the inhibition zone, MIC, protective efficiency and curative efficiency were 22.50 mm, 0.313 mg/mL, 84.49 and 79.63%, respectively.	Thiophanate-methyl (at 15 mg/mL, 15.17 mm inhibition zone, MIC = 0.313 mg/mL, protective and curative efficiency of 75.39 and 58.61%, respectively.)	[85]
**Flavone**					
Agathisflavone	*Agrobacterium tumefaciens*	In vitro	MIC value of 2 mg/mL. Antagonism was observed when combined with Kocide 3000.	Kocide 3000 (MIC = 0.13 mg/mL)	[86]
	*Erwinia carotovora* var. *carotovora*	In vitro	MIC value of 2 mg/mL. Antagonism was observed when combined with Kocide 3000.	Kocide 3000 (MIC = 0.13 mg/mL)	[86]
	*Pseudomonas corrugata*	In vitro	MIC value of 2 mg/mL. Synergism was observed when combined with Kocide 3000.	Kocide 3000 (MIC = 0.13 mg/mL)	[86]
	*Pseudomonas syringae* pv. *tomato*	In vitro	MIC value of 2 mg/mL. Synergism was observed when combined with Kocide 3000.	Kocide 3000 (MIC = 0.13 mg/mL)	[86]
	*Xanthomonas campestres* pv. *vesicatoria*	In vitro	MIC value of 2 mg/mL. Synergism was observed when combined with Kocide 3000.	Kocide 3000 (MIC = 0.13 mg/mL)	[86]
Baicalein	*Streptomyces scabiei*	In vitro	At 100 µM, bacterial cell growth was inhibited by 30%. MIC and LD_50_ values of 202.9 ± 5.3 and 52.9 ± 1.3 µM, respectively.	Novobiocin (used to establish the 0% growth baseline)	[84]
Jaceosidin	*Streptomyces scabiei*	In vitro	At 100 µM, bacterial cell growth was inhibited by 40%. MIC and LD_50_ values of 100.0 ± 2.1 and 22.6 ± 0.5 µM, respectively.	Novobiocin (used to establish the 0% growth baseline)	[84]
Isoorientin	*Agrobacterium tumefaciens*	In vitro	MIC value higher than 0.60 mg/mL.	Streptomycin sulfate (MIC = 0.04 mg/mL)	[49]
	*Pseudomonas syringae* pv. *lachrymans*	In vitro	MIC value higher than 0.60 mg/mL.	Streptomycin sulfate (MIC = 0.04 mg/mL)	[49]
	*Xanthomonas vesicatoria*	In vitro	MIC value higher than 0.60 mg/mL.	Streptomycin sulfate (MIC = 0.04 mg/mL)	[49]
Isovitexin	*Agrobacterium tumefaciens*	In vitro	MIC value higher than 0.60 mg/mL.	Streptomycin sulfate (MIC = 0.04 mg/mL)	[49]
	*Pseudomonas syringae* pv. *lachrymans*	In vitro	MIC value higher than 0.60 mg/mL.	Streptomycin sulfate (MIC = 0.04 mg/mL)	[49]
	*Xanthomonas vesicatoria*	In vitro	MIC value higher than 0.60 mg/mL.	Streptomycin sulfate (MIC = 0.04 mg/mL)	[49]
Vitexin	*Agrobacterium tumefaciens*	In vitro	MIC value higher than 0.60 mg/mL.	Streptomycin sulfate (MIC = 0.04 mg/mL)	[49]
	*Pseudomonas syringae* pv. *lachrymans*	In vitro	MIC value higher than 0.60 mg/mL.	Streptomycin sulfate (MIC = 0.04 mg/mL)	[49]
	*Xanthomonas vesicatoria*	In vitro	MIC value higher than 0.60 mg/mL.	Streptomycin sulfate (MIC = 0.04 mg/mL)	[49]
**Flavonol**					
Kaempferol	*Agrobacterium tumefaciens*	In vitro	MIC value of 0.25 mg/mL. Antagonism was observed when combined with Kocide 3000.	Kocide 3000 (MIC = 0.13 mg/mL)	[86]
		In vitro	MIC value higher than 0.60 mg/mL.	Streptomycin sulfate (MIC = 0.04 mg/mL)	[49]
	*Erwinia carotovora* var. *carotovora*	In vitro	MIC value of 2 mg/mL. Antagonism was observed when combined with Kocide 3000.	Kocide 3000 (MIC = 0.13 mg/mL)	[86]
	*Pseudomonas corrugata*	In vitro	MIC value of 0.25 mg/mL. Synergism was observed when combined with Kocide 3000.	Kocide 3000 (MIC = 0.13 mg/mL)	[86]
	*Pseudomonas syringae* pv. *lachrymans*	In vitro	MIC value higher than 0.60 mg/mL.	Streptomycin sulfate (MIC = 0.04 mg/mL)	[49]
	*Pseudomonas syringae* pv. *tomato*	In vitro	MIC value of 0.5 mg/mL. Synergism was observed when combined with Kocide 3000.	Kocide 3000 (MIC = 0.13 mg/mL)	[86]
	*Xanthomonas campestres* pv. *vesicatoria*	In vitro	MIC value of 0.5 mg/mL. Synergism was observed when combined with Kocide 3000.	Kocide 3000 (MIC = 0.13 mg/mL)	[86]
	*Xanthomonas vesicatoria*	In vitro	MIC value higher than 0.60 mg/mL.	Streptomycin sulfate (MIC = 0.04 mg/mL)	[49]
Quercetin	*Agrobacterium tumefaciens*	In vitro	MIC value of 2 mg/mL. Antagonism was observed when combined with Kocide 3000.	Kocide 3000 (MIC = 0.13 mg/mL)	[86]
		In vitro	MIC value of 0.2 mg/mL.	Streptomycin sulfate (MIC = 0.04 mg/mL)	[49]
	*Erwinia carotovora* var. *carotovora*	In vitro	MIC value of 2 mg/mL. Antagonism was observed when combined with Kocide 3000.	Kocide 3000 (MIC = 0.13 mg/mL)	[86]
	*Pseudomonas corrugata*	In vitro	MIC value of 1 mg/mL. Synergism was observed when combined with Kocide 3000.	Kocide 3000 (MIC = 0.13 mg/mL)	[86]
	*Pseudomonas syringae* pv. *lachrymans*	In vitro	MIC value of 0.4 mg/mL.	Streptomycin sulfate (MIC = 0.04 mg/mL)	[49]
	*Pseudomonas syringae* pv. *tomato*	In vitro	MIC value of 1 mg/mL. Synergism was observed when combined with Kocide 3000.	Kocide 3000 (MIC = 0.13 mg/mL)	[86]
	*Streptomyces scabiei*	In vitro	At 100 µM, bacterial cell growth was inhibited by 45%. MIC and LD_50_ values of 285.2 ± 6.8 and 37.8 ± 1.0 µM, respectively.	Novobiocin (used to establish the 0% growth baseline)	[84]
	*Xanthomonas campestres* pv. *vesicatoria*	In vitro	MIC value of 1 mg/mL. Synergism was observed when combined with Kocide 3000.	Kocide 3000 (MIC = 0.13 mg/mL)	[86]
	*Xanthomonas vesicatoria*	In vitro	MIC value of 0.2 mg/mL.	Streptomycin sulfate (MIC = 0.04 mg/mL)	[49]
Rutin	*Agrobacterium tumefaciens*	In vitro	MIC value higher than 0.6 mg/mL.	Streptomycin sulfate (MIC = 0.04 mg/mL)	[49]
	*Pseudomonas syringae* pv. *lachrymans*	In vitro	MIC value higher than 0.6 mg/mL.	Streptomycin sulfate (MIC = 0.04 mg/mL)	[49]
	*Xanthomonas perforans*	In vitro and In vivo	At 2 mM, rutin had no effect on antibacterial activity in vitro but reduced the disease severity of bacterial spot.	n.a.	[87]
	*Xanthomonas vesicatoria*	In vitro	MIC value higher than 0.6 mg/mL.	Streptomycin sulfate (MIC = 0.04 mg/mL)	[49]
**Isoflavone**					
Biochanin A	*Acidovorax citrulli*	In vitro	MIC value lower than 200 µg/mL.	n.a.	[81]
	*Clavibacter michiganensis*	In vitro	MIC value lower than 200 µg/mL.	n.a.	[81]
	*Erwinia amylovora*	In vitro	MIC value lower than 200 µg/mL.	n.a.	[81]
	*Xanthomonas axonopodis* pv. *glycines*	In vitro and In vivo	MIC value lower than 100 µg/mL. Significantly reduced the number of lesions per soybean leaf at 100, 200, and 500 µg/mL (135.3 ± 18.2, 89.8 ± 30.1, and 27.8 ± 7.7).	Chloromycetin (500 μg/mL, the number of lesions per leaf was 62.4 ± 17.9)	[81]
Genistein	*Acidovorax citrulli*	In vitro	MIC value lower than 500 µg/mL.	n.a.	[81]
	*Clavibacter michiganensis*	In vitro	MIC value lower than 500 µg/mL.	n.a.	[81]
	*Erwinia amylovora*	In vitro	MIC value lower than 500 µg/mL.	n.a.	[81]
	*Xanthomonas axonopodis* pv. *glycines*	In vitro	MIC value lower than 500 µg/mL.	n.a.	[81]
**Others**					
(3R)-2′,3′,7-trihydroxy-4′-methoxyisoflavanone	*Ralstonia solanacearum*	In vitro	Showed an inhibition zone diameter of 8.11 mm.	Streptomycin sulfate (16.80 ± 0.33 mm)	[82]
(3R)-4′-methoxy 2′,3,7-trihydroxyisoflavanone	*Ralstonia solanacearum*	In vitro	Showed an inhibition zone diameter of 9.99 mm.	Streptomycin sulfate (16.80 ± 0.33 mm)	[82]
(3R)-vestitol	*Ralstonia solanacearum*	In vitro	Showed an inhibition zone diameter of 16.62 mm.	Streptomycin sulfate (16.80 ± 0.33 mm)	[82]
(3R)-vestitone	*Ralstonia solanacearum*	In vitro	Showed an inhibition zone diameter of 11.19 mm.	Streptomycin sulfate (16.80 ± 0.33 mm)	[82]
Sativanone	*Ralstonia solanacearum*	In vitro	Showed an inhibition zone diameter of 6.53 mm.	Streptomycin sulfate (16.80 ± 0.33 mm)	[82]

Note: MIC (minimum inhibitory concentration) and LD_50_ (median lethal dose). Concentration units are reported in mass (mg/mL, μg/mL) or molar (μM) values. Note that 1 μg/mL=0.001 mg/mL; conversions between mass-based (mg/mL, μg/mL) and molar concentrations (μM) depend on the molecular weight of each individual flavonoid compound.

### 4.1. Modes of Action

#### 4.1.1. Effects on Cell Wall and Membrane Integrity

**Figure 6:**
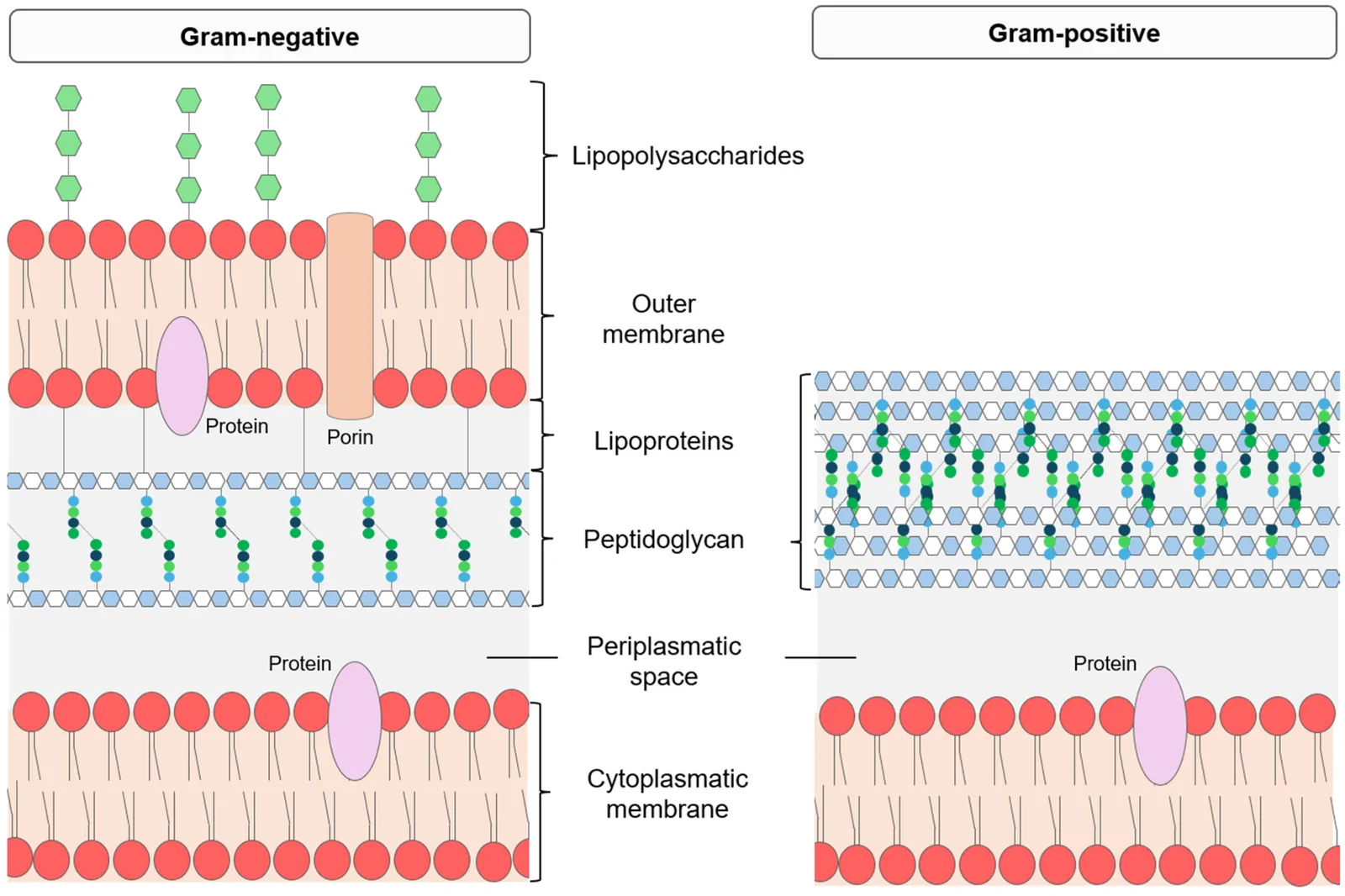
Structural organization of the bacterial cell wall and membrane.

The bacterial cell is a multilayered and dynamic structure that, among other functions, maintains cell integrity, regulates permeability, and mediates interactions with the extracellular environment. This structure organization differs between Gram-negative and Gram-positive bacteria (Figure 6), primarily due to differences in peptidoglycan layer thickness and the presence or absence of an outer membrane. In all bacteria, the inner cytoplasmic membrane is a phospholipid bilayer containing proteins responsible for nutrient transport, energy generation, and signal transduction [88]. The peptidoglycan layer surrounds this membrane, providing mechanical strength and protection against osmotic pressure. In Gram-positive bacteria, the layer forms a thick network supported by teichoic and lipoteichoic acids, while it remains thinner in Gram-negative organisms and is stabilized to the outer membrane by lipoproteins. The outer membrane of Gram-negative bacteria differs from the inner one in the presence of non-specific diffusion channel proteins called porins and lipopolysaccharides that act as barriers against external components, including antibiotics. In this type of bacteria, lipoproteins can be localized in the inner membrane or in the inner part of the outer membrane, where they can interact with the peptidoglycan layer, assisting in envelope stabilization. Between the inner and outer membranes lies the periplasmic space, a metabolically active compartment containing enzymes involved in nutrient processing, detoxification, and cell wall biosynthesis. Altogether, these structural features govern the susceptibility to antimicrobial compounds, making the cell envelope a major target for flavonoid-based antibacterial strategies.

Electron microscopy analysis of *Streptomyces scabiei*, a Gram-positive bacterium that causes potato scab as well as damage in other crops, treated with sophoraflavanone G and quercetin in vitro unveiled the presence of flat and deflated structures with hyphae fragmentations and visible expelled cell content, indicating cell death [84]. Those treated with baicalein showed uneven growth in their membranes. In another in vitro study, taxifolin reduced membrane potential, disrupted the integrity of the cell membrane, and promoted the leakage of nucleic acids and proteins of *Clavibacter michiganensis* subsp. *sepedonicus*, another Gram-positive bacterium that leads to extensive economic losses in tomato and potato cultivation [85]. The authors suggested that the hydroxyl groups from the flavanonol were responsible for these changes in membrane integrity. However, in both studies, the observed membrane disruptions represent physiological responses associated with flavonoid treatment rather than a direct molecular target, emphasizing the need for in-depth mechanistic studies.

#### 4.1.2. Effects on Biofilm Formation

Gram-positive and Gram-negative bacteria form biofilms, which consist of a protective membrane formed by microbial aggregation embedded in a self-produced matrix [89]. This hydrated surface structure is mainly composed of extracellular polymers formed of proteins, lipids, polysaccharides, and nucleic acids. Biofilms are believed to be responsible for bacterial survival, allowing for their growth in natural environments. In agriculture, these structures reduce crop yield and quality, thereby impacting the safety of plant tissues and organs [90]. Therefore, biofilm formation is a crucial target for the development of bactericides. To the best of our knowledge, information on the effects of flavonoids on biofilm formation by phytopathogenic bacteria remains scarce. Despite this, rutin, chlorogenic acid and quercetin have been reported to reduce biofilm formation on *Xanthomonas campestris* pv. *campestris* by 91, 70 and 50%, respectively, in vitro. Nevertheless, it remained unclear whether biofilm inhibiting originated from direct biofilm-related molecular targets (such as quorum sensing or adhesin synthesis) or a stress response [91].

#### 4.1.3. Effects on Motility

Most bacterial cells are motile, an ability that allows them to move toward nutrient-rich environments and supports their dispersal and colonization of plant tissues. The locomotion mechanism depends on the state of the growth medium [92]. In liquid media, bacteria can swim freely using flagella that assist their rotation, whereas in solid media, bacteria can move through different mechanisms, such as twitching through pilus retraction and sliding through cell multiplication [93]. For these reasons, bactericide-induced changes in bacterial motility have been a target of research, including the use of flavonoids such as morin and naringenin, which decrease the swimming and swarming of *Pseudomonas syringae* pv. *tomato* in vitro, but to a lesser degree than the chalcone phloretin [94]. Mechanistically, this physiological response is explained by the loss of flagella and inhibition of the type III secretion system, likely mediated by the impairment of the GacS/GacA regulatory system and subsequent depletion of rsmY RNA.

#### 4.1.4. Multisite Activity

Biochanin A is an example of the versatility of mechanisms of action by which flavonoids exert their antibacterial activity in vitro. This *O*-methylated isoflavone interferes with the membrane-related gene functions of *Xanthomonas axonopodis* pv. *glycines*, downregulating genes related to the outer membrane and efflux proteins and upregulating genes encoding membrane receptors and transporters [81]. Downregulated gene expression was also verified for three genes associated with flagella formation, three genes related to DNA synthesis, and one gene related to pathogenicity. Morphologically, 50 µg/mL Biochanin A altered cell length and shape, with treated cells being smaller than the control and adopting a rounded shape rather than a cylindrical format. At this concentration, bacterial motility, extracellular protease activity, and biofilm formation were reduced, the latter by more than 50%.

Another example of in vitro multisite-described mechanisms was made for kaempferol against *Xanthomonas oryzae*, which affected normal cell morphology, as evidenced by the presence of wrinkled surfaces, indistinct cell borders, and large cavities on treated cells, suggesting intracellular content leakage and cell death [95]. Moreover, normal microbial and energy metabolism were compromised in the treated cells, including carbon metabolism, glyoxylate and dicarboxylate metabolism, and fructose and mannose metabolism. Kaempferol also significantly affected bacterial secretion systems and quorum sensing, as evidenced by the 67.63% reduction in extracellular polysaccharides and 92.78% inhibition of biofilm formation, both at 200 µg/mL. Transcriptomic analysis of 115 differentially expressed genes showed the upregulation and downregulation of 22 and 93 genes, respectively.

### 4.2. Structure–Activity Relationship

Most studies exploring the structure–activity relationships for antibacterial assessment have explored the activity of modified flavonoids rather than their native forms to determine the best structural modification for improved activity.

**Figure 7:**
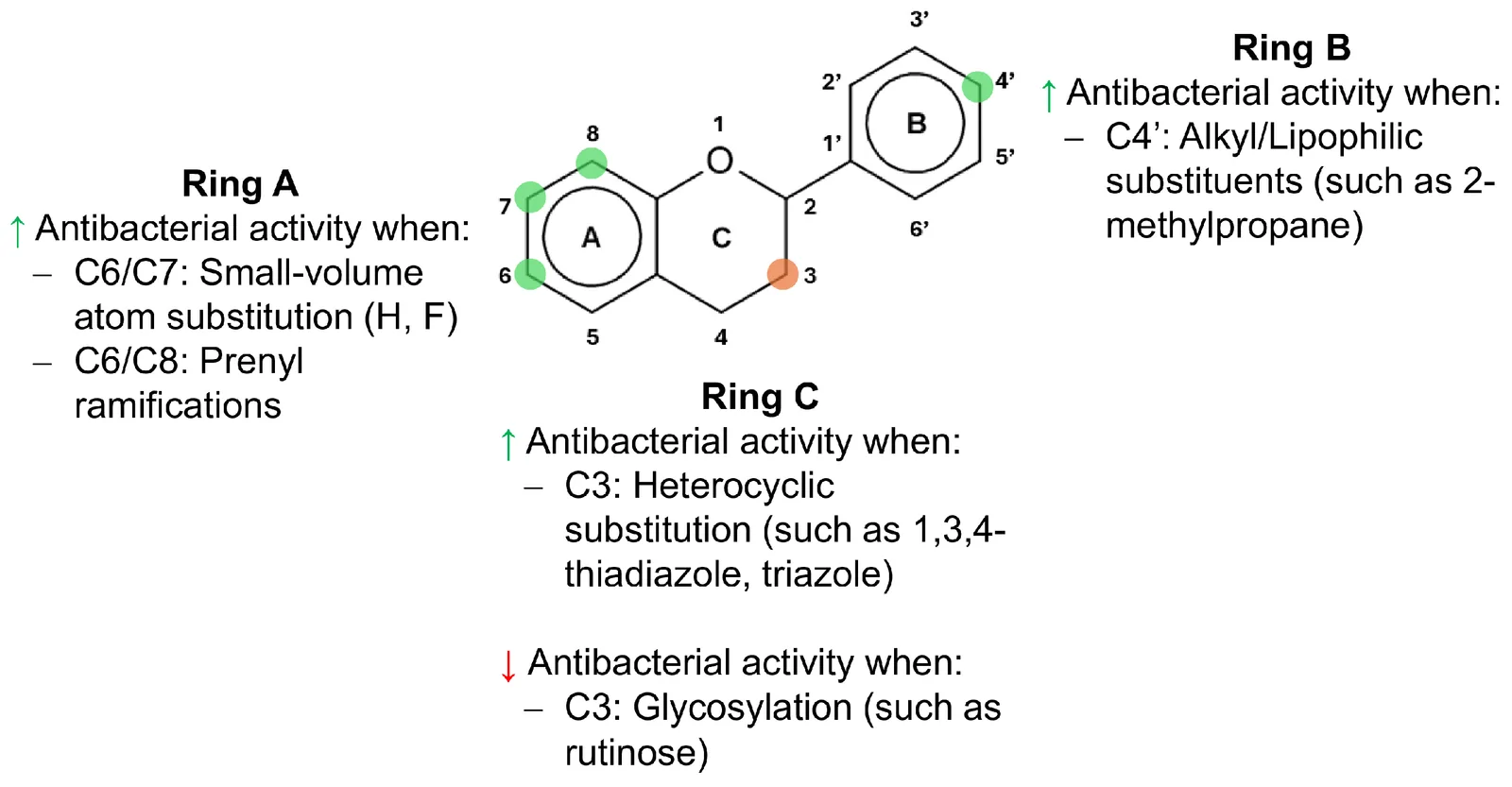
Structure–activity relationships (SAR) of native and modified flavonoids against phytopathogenic bacteria. Symbols demonstrate the impact of specific substitutions on antibacterial activity (↑: enhanced activity; ↓: reduced activity).

Antibacterial activity of flavonoids is typically increased through minimization of steric hindrance through hydrogen and/or halogen substitutions in C6, C7 and C4′ positions (Figure 7). For example, phosphorylated flavonoid derivatives (substitution in the C3 position) with hydrogen substitution at position C7 and a 2-methylpropane group at position C4′ exhibited a high antibacterial activity against *X. oryzae* pv. *oryzae* (96.09%) and *X. axonopodis* pv. *citri* (79.14%) at 100 µg/mL [96]. These observations suggest that small-volume atoms minimize steric hindrance at the C7 position and facilitate interactions with bacterial cell walls and membranes, whereas 2-methylpropane group substitution at the C4′ position increases molecule hydrophobicity, subsequently increasing membrane affinity. Similarly, substitutional changes at position C6 significantly influenced the antibacterial activity against *X. oryzae* pv. *oryzae*, with hydrogen and fluorine atom substitutions improving the antibacterial activity, likely due to reduced steric interference [97].

The integration of heterocyclic groups in the flavonoid chemical structure at, for example, position C3, is also common to achieve a more pronounced antibacterial activity. Benzimidazole-myricitrin derivatives (substitution at the C3 position) revealed that the incorporation of a 4-methylphenyl group into benzimidazole moiety resulted in the strongest antibacterial activity observed against *R. solanacearum* (EC_50_ of 14.5 µg/mL), significantly higher than myricetin (EC_50_ of 82.3 µg/mL) [98]. This pronounced activity relies on the antimicrobial properties of benzimidazole moiety, whose derivatives are commonly found in the pesticide market. Additionally, the hydrophobic properties of the 4-methylphenyl group increased lipophilicity, allowing better molecule permeation through the biological membranes.

The addition of prenyl chains to the molecule backbone is reported to increase the antibacterial activity. For example, prenylated flavonoids exhibited lower IC_50_ and MIC values than their respective non-prenylated forms against *Pseudomonas syringae*, *Agrobacterium tumefaciens*, and *Pectobacterium carotovorum* [99]. The genistein analog with prenyl substitutions at C6 and C8 showed an IC_50_ value of 3.9 µM against all tested species, whereas genistein showed an IC_50_ value higher than 250 µM. Therefore, the hydrophobic prenyl substitutions acted as lipophilic anchors, enhancing membrane interaction.

## 5. Flavonoid Formulation

Pesticide formulation plays an important role in facilitating the handling and application of pesticides and in increasing the pesticide efficiency and safety. The pesticides and other components can be transformed into solid formulations (such as dusts, granules, and wettable powders), liquid formulations (examples include oil-in-water emulsions and microcapsule suspensions), and others (like fumigants and aerosols) [100]. The chosen design directly influences the application and success of the pesticide in situ and, therefore, different formulations should be studied for a pesticide. For botanical pesticides, their formulation is crucial for a successful application in the field, given their lack of efficiency and stability as a result of their natural degradation when exposed to abiotic stress, such as light and ultraviolet radiation [20,101]. Consequently, innovative delivery strategies are being increasingly explored to overcome these issues and accelerate the acceptance of botanical pesticides as alternatives or complements to synthetic pesticides [102].

Little is still known about flavonoid formulation for in situ application, revealing a large gap in the research of these compounds as biopesticides and posing a barrier to their regulation and acceptance as plant-protecting agents. Recently, Maria et al. (2023) [103] studied the effect of the adjuvant Dassoil, a water non-ionic surface-active substance designed for agricultural use, on the MIC and MFC values of quercetin and rutin against five fungi species. The authors verified that the flavonoids formulated in Dassoil showed lower MIC and MFC values for all tested fungi than when tested alone [103]. Although Dassoil ensures good spraying quality, this solvent is considered toxic to aquatic life with long-lasting effects and can pose risks to human health if not properly managed [104]. These results show the importance of considering the interactions between biopesticides and their respective carriers without ignoring their combined toxicological profile [105].

Current trends in pesticide formulation focus on controlled-release systems, which allow the use of lower amounts of pesticide through their sustained release and target delivery to the pest surface or through permeation of their biological membrane [106]. These characteristics contribute to the decrease in the risk of unintended exposure of animals and the environment. Nanoporous materials are commonly used as encapsulating materials for this purpose since they present a high surface area and porosity, and their surface can be tailored to improve certain characteristics, such as water solubility, stability, and encapsulation loading [107]. Particularly, this type of formulation has been studied for flavonoids and has shown promising results. Kaempferol-loaded lecithin/chitosan nanoparticles exhibited higher antifungal activity than pure kaempferol against *Fusarium oxysporum* in vitro, and microscopic observations of hyphae revealed distortions and fragmentations that were absent in pure kaempferol-treated hyphae [47]. The authors assumed that the small and compact features of the nanoparticles, combined with their high surface area, resulted in greater absorption onto the surface of the fungal cell wall, leading to higher activity. In another study, didymin and a flavonoid-rich orange peel extract encapsulated in chitosan nanoparticles completely inhibited the in vitro mycelial growth of *A. alternata* after 7 days of incubation, performing similarly to the unloaded chitosan nanoparticles [108]. Against *P. expansum* and *Aspergillus westerdijkiae*, these nanoparticles showed a higher mycelial inhibition than didymin, the flavonoid extract, and the unloaded chitosan nanoparticles. Furthermore, in vivo studies performed on pear fruits revealed a more pronounced decrease in lesion diameter on all tested fungal species for the loaded nanoparticles compared to both the negative control and unloaded chitosan nanoparticles.

In addition to these nanoporous systems, metallic nanoparticles offer a distinct controlled-release pesticide system owing to their high surface reactivity and bioavailability, which has been studied in flavonoid formulations. The elements typically chosen are essential for plant development and germination, such as magnesium and zinc, and, if not, they possess antimicrobial properties, such as silver [109,110]. In particular, naringin self-assembled silver nanoparticles exhibited a significantly lower IC_50_ value against *Penicillium glabrum* compared to the silver nanoparticles alone (21.01 and 59.22 µg/mL, respectively) and disrupted cell membrane integrity, induced lipid droplet aggregation, and regulated the expression of ROS-generating related genes [111].

To overcome the inherent physicochemical limitations of flavonoids (particularly poor water solubility and susceptibility to abiotic degradation), cyclodextrin-based delivery systems could help overcome these issues. Cyclodextrins are non-reducing cyclic oligosaccharides able to form stable and water-soluble inclusion complexes with flavonoids, which have been widely used in the biomedical field to overcome solubility and bioavailability issues [112]. However, to the best of our knowledge, these systems have not been considered for delivery of flavonoids as antimicrobial pesticides.

In botanical pesticide research, nanoemulsions are often reported for essential oil encapsulation, solving issues such as degradation and volatility [23]. For flavonoids, however, this form of encapsulation is uncommon because standard oil-in-water nanoemulsions are designed for highly lipophilic compounds and, therefore, applying them to flavonoids could result in their precipitation or agglomeration. Consequently, current applications of nanoemulsions with flavonoids are limited to co-formulation strategies where these polyphenols are incorporated within essential oil nanoemulsions to enhance activity. For example, eugenol nanoemulsion loaded with nobiletin showed a lower MIC value than the unloaded eugenol nanoemulsion against *P. italicum* (160 and 320 µg/mL, respectively) [113]. This observation was also reflected in a higher mycelial growth inhibition of nobiletin-loaded eugenol nanoemulsions than of unloaded nanoemulsions (54.68 and 9.92%, respectively). Additionally, in vitro assays revealed that the nobiletin-loaded nanoemulsions caused more morphological damage to the cell structure and possessed better release control and effectively delayed disease fungal growth and reproduction in citrus fruits than the unloaded nanoemulsions. In an in vitro study, citral nanoemulsions containing polymethoxylated flavonoids decreased the total lipid and ergosterol contents in *P. italicum*, along with an increase in extracellular electrical conductivity and fluorescence intensity of propidium iodide in a concentration-dependent manner, evidencing membrane damage [114].

Similarly, the literature research revealed that lipid-based systems, such as liposomes, have not been considered as nanocarriers for flavonoid delivery to the surface of fungi or bacterial species. This lack of consideration lies heavily on the economic costs associated with liposomal formulations (for example, expensive purification steps and multi-step manufacturing processes) and poor physical and chemical stability under field conditions, being susceptible to oxidation and premature thermal degradation [115].

## 6. Flavonoids Position Within the Biopesticide Sector

The growing international pressure for more sustainable agricultural practices, triggered by consumer demand for organically produced food and governmental restrictions on synthetic pesticides, has significantly accelerated the growth of the biopesticide market. According to Verified Market Research, the biopesticide sector was valued at USD 6.66 billion in 2024 and is expected to reach USD 17.09 billion by 2032, expanding at a Compound Annual Growth Rate (CAGR) of 13.8% between 2026 and 2032 [116]. Within this market, the botanical pesticide segment alone reached USD 1.5 billion in 2025, with an estimated CAGR of 9.1% from 2026 to 2034 [117]. These predictions align with the rise in published research on this topic, with half of the 5054 publications between 1994 and 2024 being published in the last five years [118]. The growth of scientific output has also contributed to an increase in patent applications and registrations related to crop protection, including those focused on flavonoids as antimicrobial pesticides (Table 3).

In the EU, potential flavonoid-based antimicrobial pesticides are governed by the Regulation (EC) No 1107/2009 [125] and Directive 2009/128/EC [126]. The Regulation (EC) No 1107/2009 addresses the authorization, commercialization, and use of plant protection products, including pesticides and biopesticides [125]. However, the current regulation does not define botanical pesticides clearly and, consequently, problems are detected throughout the authorization process. For instance, Vekemans and Marchand (2020) verified that chemical pesticides with similar toxicological data were approved, whereas other botanicals were not [127]. The authors also observed that no botanical low-risk pesticide was approved or considered, despite 30 promising applications from 2007 to 2019. This regulation drawbacks contradict one important principle of the Directive 2009/128/EC, which states that “Sustainable biological, physical and other non-chemical methods must be preferred to chemical methods if they provide satisfactory pest control” (ANNEX III, Principle 4) [126]. As a result of legislation inconsistencies, flavonoid-based antimicrobial pesticides could face substantial difficulties for market integration in the EU.

To overcome these issues, global harmonization standards would allow uniformized information and accelerate their commercialization. As a matter of fact, the Food and Agriculture Organization of the United Nations (FAO) and World Health Organization (WHO) published an International Code of Conduct on Pesticide Management and specific registration guidelines for biopesticides, including data requirements for botanical pesticides [128]. More specifically, the data requirements consist of intended use; identity, characterization, physical, chemical, biological properties and analytical methods; human health; residues; and environment, ecotoxicology, and efficacy. Additionally, the guidelines clearly mention that the term “botanical” can cover a heterogeneous group of molecules, from plant powders to unprocessed plant extracts and refined botanicals such as one single active molecule. If the EU were to align with these guidelines, flavonoid-based antimicrobial pesticides could be approved and integrated into the biopesticide market.

Farmer’s acceptance and application of flavonoid-based antimicrobial pesticides will also dictate their successful integration into the biopesticide market and into microbial pathogen management practices. Current barriers for botanical pesticides are cost-effectiveness and market competitiveness compared to synthetic pesticides or other commercialized botanical pesticides, perceived inconsistent efficiency, slow transition from conventional to sustainable agricultural practices, and lack of farmers’ knowledge regarding botanical pesticides and their correct application. For example, 20% of 202 vegetable farmers in Bangladesh had good knowledge of botanical pesticides but 43% were willing to adopt them, probably as a result of rising consumer demand for organic products [129]. In the Mediterranean Region, farmers in countries such as Spain, Tunisia, and Turkey face economic and informational barriers, along with skepticism and common conventional practices [130]. As a result, farmer education through training programs (including in the homemade preparation of botanical pesticides) and cooperative support can facilitate their adoption.

## 7. Conclusions and Future Perspectives

Flavonoids have emerged as promising antimicrobial pesticides for the management of pre- and postharvest phytopathogens, being compelling complements to conventional pesticides. The literature consistently shows that these polyphenols display antifungal and antibacterial properties, often acting through multiple mechanisms of action, which is a crucial feature for reducing the risk of resistance development. Despite these strengths, the integration of flavonoids into pest and disease management remains challenging. Botanical pesticides are often described as complements to traditional pesticides rather than complete alternatives owing to their lack of field efficiency as a consequence of natural degradation. Although the formulation of these compounds can improve their efficiency, the correct formulation for field applications may take a long time to discover and adjust to the specific target, compromising the entry of flavonoids in the biopesticide sector. However, the future of flavonoids in this sector could also rely on studying synergistic and additive effects between these molecules and traditional pesticides on the market, other classes of antimicrobial compounds, or their respective carriers or solvents. A recent study highlighted the synergistic effects of three flavonoids (quercetin, kaempferol, and agathisflavone) and Kocide 3000 against *Pseudomonas corrugata*, *P. syringae* pv. *tomato*, and *Xanthomonas campestres* pv. *vesicatoria*, whereas additive effects were detected for *P. carotovora* and *A. tumefaciens* [86]. Combined with optimized formulations and mechanistic elucidation, this perspective could redirect the study of these molecules, resulting in promising and innovative flavonoid-based bioproducts for pest management and contributing to the overall reduction of traditional pesticide applications.

To accelerate this transition and build knowledge, future research on flavonoid-based antimicrobial pesticides must make use of modern technological tools. Multi-omics approaches (such as genomic, transcriptomic, proteomic, and metabolomic) allow a more in-depth mechanistic comprehension and a deeper understanding of plant–pathogen–flavonoid interaction, especially under environmental stress conditions [131]. When combined with machine learning, artificial intelligence and molecular design precision, these tools could facilitate the design of new and optimized flavonoid structures for specific molecular targets [132,133]. Additionally, these computational models have been used to predict synergistic interactions of different formulations of botanical pesticides and permeation characteristics, such as essential oils of *Thymus* spp. against *Musca domestica* L [134,135], as well as pesticide toxicity [136]. Therefore, these advanced technological tools and models can help transition laboratory knowledge on flavonoids as antimicrobial pesticides into effective agriculture field applications.

## Figures and Tables

**Table 3:** Examples of registered patents of flavonoids as antimicrobial pesticides.

Compounds	Phytopathogen	Application Patent Number
**Bacteria**		
5 flavonoids (7,8-dihydroxyflavone, baicalein, scutellarin, glycyrrhizin and naringenin)	*Pseudomonas syringae* pv. *actinidiae* *Xanthomonas oryzae* pv. *oryzicola*	CN 118285385A [119]
44 flavonoids (including baicalein and scutellarein)	*Xanthomonas oryzae* *Xanthomonas axonopodis* pv. *citri*	CN 113455507A [120]
**Fungi**		
Hyperoside Trifolin	*Alternaria alternata* *Epicoccum nigrum* *Pestalotia quepinii* *Drechslera* sp. *Fusarium avenaceum*	US 2007/0134282 [121]
5,7,4′-tri-O-flavanone 5,7,3′4′-tetra-O-acetylflavanone	*Fusarium oxysporum*	US 2009/0258097 [122]
kaempferol-3-0-α-rhamnopyranosyl-(1-2)-β-glucopyranoside	*Aspergillus niger*	WO 2018/048296 [123]
Karanja Oil Flavonoid Extract + Saturated Aliphatic Acids	*Botrytis cinerea*	WO 2019/064283 [124]

## Data Availability

No new data were created or analyzed in this study. Data sharing is not applicable to this article.

## Abbreviations

The following abbreviations are used in this manuscript:

IC_50_	Half maximal inhibitory concentration
MIC	Minimum inhibitory concentration
MFC	Minimum fungicidal concentration
LD_50_	Mean lethal dose
ROS	Reactive oxygen species
MDA	Malondialdehyde
ATP	Adenosine triphosphate
ADP	Adenosine diphosphate
AMP	Adenosine monophosphate
DNA	Deoxyribonucleic acid
